# Electrophoretic cytopathology resolves ERBB2 forms with single-cell resolution

**DOI:** 10.1038/s41698-018-0052-3

**Published:** 2018-03-22

**Authors:** Chi-Chih Kang, Toby M. Ward, Jessica Bockhorn, Courtney Schiffman, Haiyan Huang, Mark D. Pegram, Amy E. Herr

**Affiliations:** 1 Department of Bioengineering, University of California Berkeley, Berkeley, CA 94720 USA; 2 Division of Medical Oncology, Department of Medicine, Stanford University, Stanford, CA 94305 USA; 3 Division of Biostatistics, School of Public Health, University of California Berkeley, Berkeley, CA 94720 USA; 4 Department of Statistics, University of California Berkeley, Berkeley, CA 94720 USA

## Abstract

In addition to canonical oncoproteins, truncated isoforms and proteolysis products are implicated in both drug resistance and disease progression. In HER2-positive breast tumors, expression of truncated HER2 isoforms resulting from alternative translation and/or carboxy-terminal fragments (CTFs) resulting from proteolysis (collectively, t-erbB2) have been associated with shortened progression-free survival of patients. Thus, to advance clinical pathology and inform treatment decisions, we developed a high-selectivity cytopathology assay capable of distinguishing t-erbB2 from full-length HER2 expression without the need for isoform-specific antibodies. Our microfluidic, single-cell western blot, employs electrophoretic separations to resolve full-length HER2 from the smaller t-erbB2 in each ~28 pL single-cell lysate. Subsequently, a pan-HER2 antibody detects all resolved HER2 protein forms via immunoprobing. In analysis of eight breast tumor biopsies, we identified two tumors comprised of 15% and 40% t-erbB2-expressing cells. By single-cell western blotting of the t-erbB2-expressing cells, we observed statistically different ratios of t-erbB2 proteins to full-length HER2 expression. Further, target multiplexing and clustering analyses scrutinized signaling, including ribosomal S6, within the t-erbB2-expressing cell subpopulation. Taken together, cytometric assays that report both protein isoform profiles and signaling state offer cancer classification taxonomies with unique relevance to precisely describing drug resistance mechanisms in which oncoprotein isoforms/fragments are implicated.

## Breast cancer: A precision test for truncated oncoproteins in single cells

A single-cell western blot provides a simple way of studying whether a cancer-related protein found in breast tumors has transcriptionally or post-translationally modified to a truncated form, which is implicated in resistance to targeted therapies. The microfluidic assay, developed by Amy E. Herr from the University of California, Berkeley, USA, and colleagues at Stanford University, involves first dissociating a breast tumor, then isolating each tumor cell in cell-sized well on a microscope slide. Isolated in the well, each cell is broken up and HER2 proteins are size-separated via single-cell electrophoresis, before being immobilized and finally detected with a fluorescent probe that binds to both the full-length and truncated HER2 proteins. Herr’s team assayed eight patient samples and identified two tumor samples with mixed populations of full-length and truncated HER2 proteins, a finding with therapeutic and prognostic relevance for patients.

## Introduction

Oncoproteins and their truncated protein forms are implicated in tumor progression, metastasis, and drug resistance.^1–3^ Human epidermal growth factor receptor 2 (HER2, a.k.a. erbB2, Uniprot P04626) can be expressed as the full-length receptor or as truncated forms (t-erbB2s).^1^ Truncated HER2 oncoprotein forms arise from metalloprotease-mediated shedding yielding membrane bound or cytoplasmic carboxy-terminal fragments (CTFs),^4^ alternative initiation of translation^5,6^ or RNA splicing variants.^7^ Full-length HER2 is amplified in 15–20% of invasive breast cancers. The canonical full-length protein is targeted by trastuzumab, pertuzumab—both humanized IgG1 monoclonal antibodies, and by the antibody-drug conjugate ado-trastuzumab emtansine (T-DM1).^8^ No FDA-approved antibody-based therapies against HER2 target the truncated HER2 isoforms or CTFs.^9,10^ Neither trastuzumab, pertuzumab, nor T-DM1 can bind to t-erbB2s as the HER2 isoforms lack the extracellular domain of full-length HER2, which includes the therapeutic antibody-binding epitopes. Consequently, the expression of t-erbB2 proteins (p95, p110, or Δ16) in cancer cells suggests one possible resistance mechanism against antibody-based anti-HER2 therapies.^1,7^ Some, though not all,^11^ clinical studies have shown that metastatic HER2-positive patients expressing t-erbB2s have worse clinical outcomes when treated with trastuzumab,^12^ as evidenced by shorter progression-free survival rates.^13,14^ Comparison was made to patients expressing only full-length HER2. Moreover, expression of t-erbB2s has been associated with lymph node and brain metastases.^12,15,16^ While patients with t-erbB2s may have worse progression-free survival under anti-HER2 trastuzumab therapy, these patients can benefit from other treatments, such as erbB2-selective tyrosine kinase inhibitors.^17,18^ Consequently, precise tumor classifications that include information about expression of truncated oncoprotein isoforms and CTFs— rare tumor markers—hold promise in guiding treatment decisions for specific patients.^19^

Cytology assays capable of resolving full-length HER2 (p185HER2) and truncated (t-erbB2) oncoprotein exist, but are fraught with limitations. Immunohistochemistry (IHC, including HER2-IHC) is powerful, but requires antibodies that are specific to each protein target. Further, IHC is semi-quantitative and suffers from lab-to-lab performance variation and reproducibility concerns.^20^ To address analytical limitations, microfluidic tissue processing has made quantification of HER2 possible,^21^ with the caveat that the assay is limited to available immunoprobes (e.g., pan-HER2 detection). Objective analysis and interpretation of immunohistochemical slides benefit from machine learning approaches, but IHC assays cannot readily identify t-erbB2.^22^ Advanced spectroscopic techniques achieve high accuracy as cytology-based cancer diagnostics, but cannot provide molecular information.^23^ Single-cell targeted DNA analysis^24^ and single-cell RNA sequencing^25^ are suitable for studying genomic heterogeneity and different RNA splice variants, respectively, but cannot detect fragments produced from protein shedding. Targeted protein assays such as imaging mass cytometry^26^ are inherently immunoassays and—even with 32-target multiplexing power—are unable to detect isoforms lacking isoform-specific antibodies. The proximity-based VeraTag p95 IHC assay does selectively report expression of t-erbB2 (primarily HER2 CTF611; a.k.a. p110) in formalin-fixed, paraffin-embedded (FFPE) clinical samples.^13,15^ Given the chemical readout mechanism, the VeraTag p95 assay is unable to simultaneously measure t-erbB2 forms and full-length p185HER2 in the same cell.^27^

Clinical trial data using different t-erbB2 measuring methods suggest different lapatinib treatment responses among patients with t-erbB2 expression as compared to non-t-erbB2 expressing patients.^11,17,18^ The limitation in t-erbB2 measurement stymies t-erbB2-based clinical diagnostics.

Here, we introduce a single-cell resolution western blot (scWB)^28–30^ to assess p185HER2 and t-erbB2s in heterogeneous HER2-positive breast tumor biopsies with high specificity. In a manner similar to conventional western blots, the scWB uses electrophoresis to size-separate t-erbB2s (~90–115 kDa) from p185HER2 (185 kDa). The t-erbB2 scWB does not require isoform-specific antibody probes. Microfluidic cell and protein handling and photo-initiated protein immobilization provides sufficient sensitivity for single-cell t-erbB2 detection. Important to elucidating t-erbB2-related drug resistance signaling, protein multiplexing is accomplished by chemical stripping and re-probing of the device. The scWB slides are suitable for long-term storage,^27^ making retrospective analyses of clinical samples possible, as new hypotheses develop regarding t-erbB2 pathways.

## Results

### Single-cell polyacrylamide gel electrophoresis (PAGE) resolves t-etbB2 from p185HER2

**Fig. 1:**
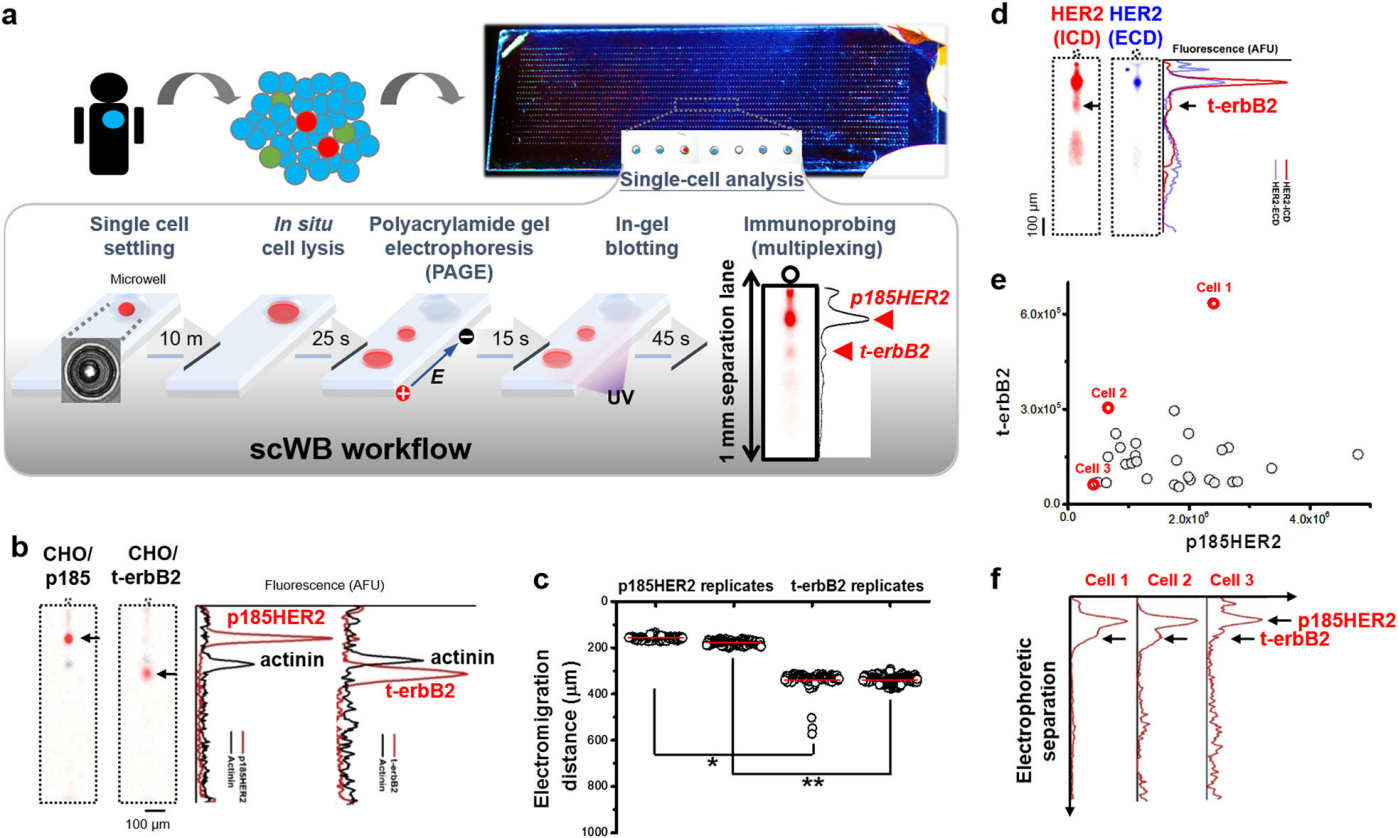
Single-cell western blotting differentiates truncated isoforms/CTFs from full-length HER2 protein in primary HER2-positive breast tumor biopsies. **a** The microfluidic scWB device is designed to assay the expression of different HER2 forms in heterogeneous tumor cells derived from dissociated HER2-positive breast cancer tumors. The scWB workflow includes five steps: gravity-based settling of single cells into each of an array of microwells, in situ chemical cell lysis, polyacrylamide gel electrophoresis (PAGE) of each single-cell lysate, UV-activated protein immobilization, and finally in-gel immunoprobing. With PAGE, the scWB can resolve truncated (t-erbB2s) from full-length HER2 oncoproteins (p185HER2) in each 1-mm long separation distance. PAGE physically separates background signal from the target-protein peaks. **b** False-color fluorescence micrographs and corresponding intensity plots resolve smaller t-erbB2s (~95 kDa) from larger p185HER2 oncoproteins (~185 kDa) in positive controls cells (genetically modified CHO cells; CHO/p185 and CHO/t-erbB2; 15-sec PAGE in 7%T, 2.6%C gel). Immunoprobing of actinin is an internal electromigration control. AFU Arbitrary Fluorescence Unit. **c** Dot plot shows a significant difference in the mean electromigration distance for p185HER2 versus t-erbB2s (two-tailed *t*-test, *p*_replicate 1_ < 1 × 10^−10^, labeled as *; *p*_replicate 2_ < 1 × 10^−^^10^, labeled as **). All measurements outside 4 times the standard deviation from each mean were discarded. **d**, False-color fluorescence micrographs and intensity plots show that HER2-2G11 antibody, which recognizes the ECD of HER2, detects the p185HER2 protein peak, but not the t-erbB2 protein peak detected using HER2-3B5 immunoprobing of the same BT474 cells. **e** and **f** Scatter plots and intensity plots of relative p185HER2 and t-erbB2 expression in a population of BT474 cells

The scWB device (Fig. 1a) is a microscope slide coated with a thin layer of photoactive polyacrylamide gel (PAG). The 1-mm single-cell PAGE lane of the scWB allows ~10^3^ concurrent single-cell western blots on each microscope slide, providing 90% probability to capture at least five cells from rare cell subpopulations (e.g., 1% t-erbB2-expressing cells from all tumor cells). We first sought to develop a scWB assay suitable for resolving high molecular mass proteins (>130 kDa) from median molecular mass proteins (~66 kDa) in the same cell.^29^ We selected a 7%T PAG to allow sufficient electro-injection of the >130 kDa p185HER2 protein from the microwell into the sieving matrix and sufficient separation of p185HER2 from smaller t-erbB2s (e.g., truncated p95 proteolysis product and p110 isoform) in the separation distance. As positive controls, we employed Chinese Hamster Ovary (CHO) cells with genetically engineered human p185HER2 (CHO/p185) and p95HER2 (CHO/t-erbB2). For scWB, we employed a pan-HER2 antibody (HER2-3B5) to detect all HER2 forms (p185HER2 and t-erbB2s). HER2-3B5 binds at the intracellular domain of HER2. Within the same electrophoresis time (15 s) and gel density (7%T PAG), t-erbB2 (p95 protein) electromigrates more quickly than p185HER2 (Fig. 1b), which is consistent with expected relative molecular masses and conventional western blot (Supplementary Fig. 1). To quantitatively evaluate the separation performance of p185HER2 and t-erbB2, we analyzed the separation resolution (Rs), defined as the peak-to-peak displacement normalized by average peak width. The t-erbB2 peak is nearly fully resolved from the p185HER2 peak (Rs = 0.93 ± 0.09 (s.d.), *n*_CHO/t-erbB2_ = 273 cells; *n*_CHO/p185HER2_ = 211 cells; Fig. 1c). While baseline resolution (Rs = 1.5) of t-erbB2 from p185HER2 is possible (using a longer PAGE duration), the 15-sec duration used here constrains electromigration within the 1-mm separation distance for even the small molecular mass HER2-related signaling proteins (i.e., extracellular-signal regulated kinase; ERK; 44 kDa and phospho-ribosomal S6; p-rs6; 32 kDa). Additionally, long electrophoresis durations may lead to diffusive protein losses during PAGE, resulting in undetectable t-erbB2 protein signal.

### scWB identifies a rare t-erbB2-expressing subpopulation

Naturally occurring t-erbB2s are present in cultured HER2-positive breast cancer cell lines (i.e., BT474 and SKBR3).^31,32^ Owing to the link between t-erbB2 expression and poor clinical outcomes, signal transduction studies suggest activation of both PI3K/AKT/mTOR and mitogen-activated protein kinase (MEK/ERK) signaling pathways upon t-erbB2 expression (mainly the p110 isoform, not other CTFs).^1,6,32,33^ Moreover, our previous studies observed oncogenic potential and induced tumor formation in transgenic mice only from the p110 isoforms, and not the full-length HER2 (p185HER2) and other shorter CTFs (648, 676, 687).^32^ Notably, these signaling studies did not scrutinize parental cell lines or clinical tissues, but studied genetically engineered cell lines (i.e., MCF-7 and T47D p95HER2 stable clones,^33^ MCF-7 expressing different carboxyl terminal fragment of HER2,^6^ human breast epithelial cells with t-erbB2 expression^32^). Consequently, we present direct, simultaneous, quantitative measurement of t-erbB2 and p185HER2 in single BT474 cells by scWB. We identified t-erbB2 and p185HER2 in individual BT474 cells (L_p185HER2_: 99.3 ± 15.7 (s.d.) μm, FWHM_p185HER2_: 93.7 ± 22.0 (s.d.) μm, *n* = 391 cells, L_t-erbB2_: 188.2 ± 18.1 (s.d.) μm, FWHM_t-erbB2_: 120.7 ± 30.0 (s.d.) μm, Rs = 0.46 ± 0.16 (s.d.), *n* = 29 cells from four independent scWB chips). After Gaussian-fitting the t-erbB2 protein peaks, we observed statistically significant difference in electromigration distance, as compared to the larger p185HER2 protein (paired *t*-test, one-tailed, *p* < 1 × 10^−10^, *n* = 29 cells). To calculate the corresponding molecular mass of t-erbB2, we generated a Ferguson plot (Supplementary Fig. 2) using known molecular mass proteins (mTOR, p185HER2, actinin, β-Tubulin, and GAPDH). The calculated molecular mass of t-erbB2 is 128.7 ± 11.5 kDa (104–160 kDa, 90% CI), matching with p110 isoforms defined from conventional western blots.^6^ Notably, no detectable t-erbB2s electromigrated slower than actinin (100 kDa) in BT474 cell lysate (Supplementary Fig. 3), suggesting no detectable p95HER2 fragments.

Further, we validated the t-erbB2s by immunoprobing the scWB with the HER2-2G11 antibody which recognizes the ECD of HER2. The HER2-2G11 immunoprobing was hypothesized to detect p185HER2 but not t-erbB2s in t-erbB2-expressing BT474 cells. Through stripping and re-probing of the blotting gel now using the HER2-2G11 antibody, we observed only p185HER2 signal (Fig. 1d), thus validating the higher mobility protein forms recognized by the HER2-3B5 antibody as the t-erbB2s.

**Fig. 2:**
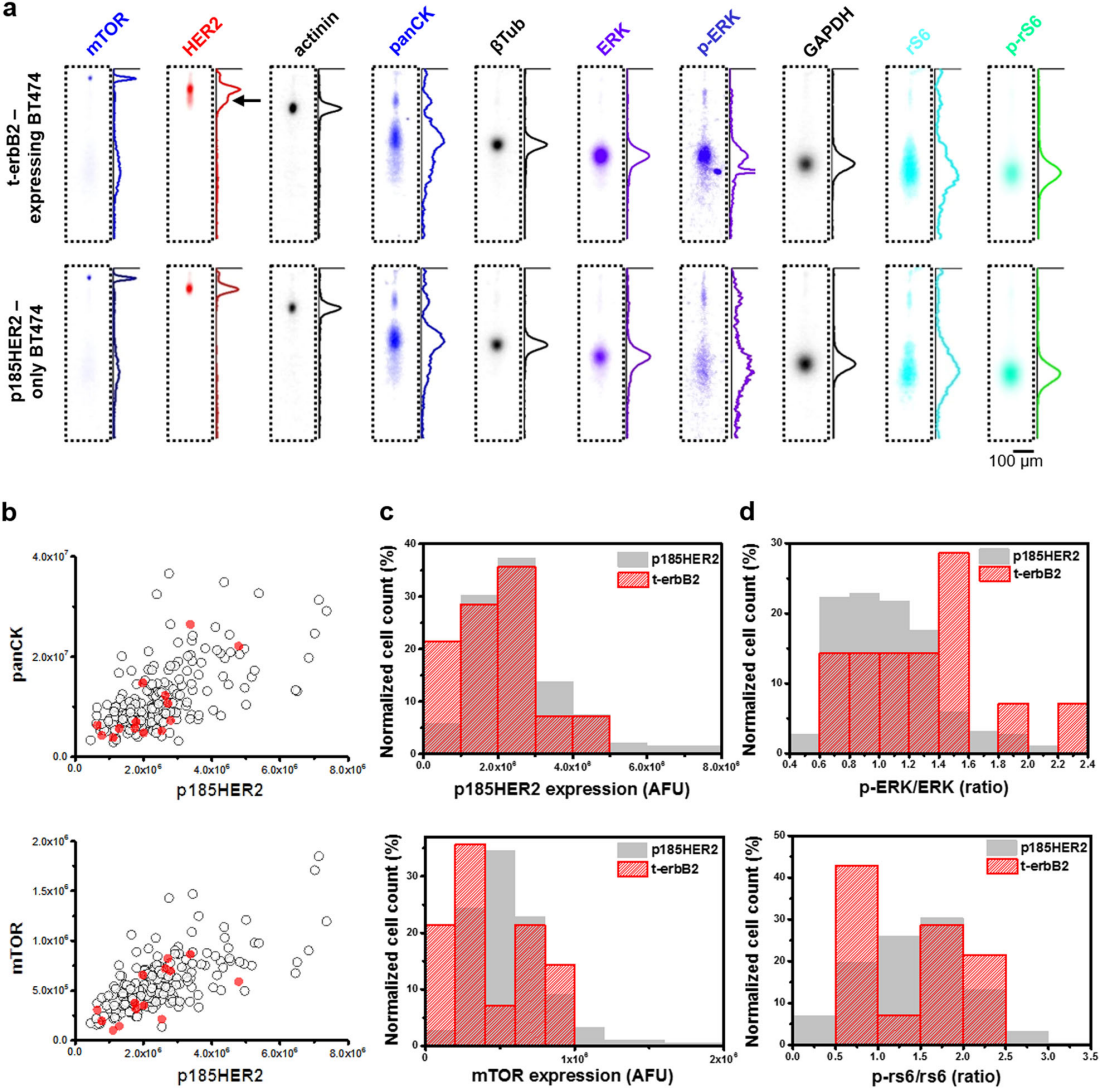
Protein profiling t-erbB2-expressing and p185HER2-only BT474 cells. **a** False-color fluorescence micrographs and intensity plots of control proteins (GAPDH, actinin and β-Tubulin), HER2-related signaling proteins (HER2, mTOR, ERK, p-ERK, rs6, and p-rs6), and an epithelial cell protein marker (panCK) are shown in a selected t-erbB2-expressing BT474 cell (top) and p185HER2-only BT474 cell (bottom). The arrow indicates the t-erbB2 protein peak. **b** The scatter plots of p185HER2 and panCK (top) and p185HER2 and mTOR (bottom) suggest that all the HER2-positive BT474 cells are panCK-positive and mTOR-positive. (○) denotes p185HER2 only BT474 cells. (●) denotes t-erbB2-expressing BT474 cells. **c** The histograms of p185HER2 (top) and mTOR (bottom) expression indicate a similar expression range between t-erbB2-expressing and p185HER2-only BT474 cells. **d** The histograms of p-ERK to ERK ratios (top) and p-rs6 to rs6 ratios (bottom) indicate the signal activation of t-erbB2-expressing versus p185HER2-only BT474 cells. The cell number of each bin is normalized to the total cell number

In this population, we identified 29 t-erbB2-expressing cells (7.4%) among 391 HER2-positive BT474 cells. Intriguingly, we found t-erbB2 proteins always co-exists with p185HER2 in an individual cell, suggesting that both t-erbB2 proteins and p185HER2 contribute to signal transduction in the t-erbB2-expressing cells. While the p185HER2 expression in t-erbB2-expressing BT474 cells has no statistically significant expression level difference from that of the p185HER2-only BT474 cells (Mann-Whitney *u*-test, two-tailed, *p*_p185HER2_ = 0.144, *n*_p185HER2_ = 188 cells, *n*_t-erbB2_ = 14 cells) (Fig. 2), the cell-to-cell t-erbB2 protein expression varied (CV = 77%). Figure 1 e, f reports representative cells spanning the spectrum from high t-erbB2 expression (cell 1), high t-erbB2s to p185HER2 expression ratios (cell 2), to low t-erbB2s expression (cell 3). Assuming 5 × 10^5^ p185HER2 receptors per BT474 breast cancer cell,^34^ we estimate between 1 × 10^4^ and 2.5 × 10^5^ copies of t-erbB2 proteins per cell. Further, given the small size of the t-erbB2-expressing cell subpopulation, we estimate total t-erbB2s at 160 femtograms, thus highlighting the challenge of measuring t-erbB2s with conventional western blotting of pooled cell populations.

### Multiplexed scWB profiles t-erbB2-related signaling

Next, we sought to investigate signal transduction in individual t-erbB2-expressing BT474 cells. In each cell, we assayed a total of 10 unique protein targets (mTOR, HER2, actinin, pan-cytokeratin (panCK), β-Tubulin (βTub), ERK, phospho-ERK (p-ERK), GAPDH, rs6, and phospho-rs6 (p-rs6)) using 6 rounds of stripping and reprobing (Fig. 2a). Among the 10 targets, p-rs6 (Ser240/Ser244) served as a robust biomarker for PI3K/AKT/mTOR signal activation^35^ and p-ERK (Thr202/Tyr204) served as a marker for MAPK signal activation.^6,33^ As expected, all HER2-positive BT474 cells were panCK-positive (Fig. 2b). Moreover, the internal control targets (actinin, GAPDH and βTub) were all strongly positively correlated (Supplementary Fig. 4). In the analysis of mTOR protein, we observed monotonic relationships between HER2 and mTOR (Spearman’s Rank Correlation ρ_HER2-mTOR_ = 0.65, *p* = 5 × 10^−7^, *n* = 188 cells), supporting the involvement of mTOR in HER2-related signaling (Fig. 2b).

Furthermore, we compared the signaling between t-erbB2-expressing and p185HER2-only BT474 cells (Fig. 2c,d). The mean expression of mTOR between t-erbB2-expressing and p185HER2-only BT474 cells is not statistically different (Mann-Whitney *u*-test, two-tailed, *p*_mTOR_ = 0.095, *n*_p185HER2_ = 188 cells, n_t-erbB2_ = 14 cells). Moreover, the p-rs6 to rs6 ratio between t-erbB2-expressing and p185HER2-only BT474 cells is also not statistically different (Mann-Whitney *u*-test, two-tailed, *p*_p-rs6/rs6_ = 0.99, n_p185HER2_ = 188 cells, *n*_t-erbB2_ = 14 cells). Together, this result implied a similar activation profile in PI3K/AKT/mTOR signaling. In contrast, we observed a slight upshift of the p-ERK to ERK ratio in t-erbB2-expressing BT474 cells compared to p185HER2-only BT474 cells (Mann-Whitney *u*-test, two-tailed, *p* = 0.038, *n*_p185HER2_ = 188 cells, *n*_t-erbB2_ = 14 cells). Notably, there is one t-erbB2-expressing cell exhibiting a high p-ERK to ERK ratio compared to the p185HER2-only cells (empirical *p*-value = 1/188 = 0.005). Taken together with the calculated t-erbB2 molecular mass, the expression of t-erbB2s (e.g., p110 isoforms) in HER2-positive breast cancer cells may enhance activation of MAPK signaling, supporting a role of t-erbB2s in drug resistance. Similar observation of p110 isoform-induced signal activation have been reported in p110/MCF-7^6^ or p110/HMLE cells.^32^ The scWB assay described here was developed to resolve full-length HER2 protein from t-erbB2 protein forms, with future assay development focused on detecting the presence of the various specific t-erbB2 protein forms, in combination with measurement of the downstream signaling events. Building off of the foundational single-cell HER2 assay, future studies regarding trastuzumab-based and HER2-selective tyrosine kinase inhibitor-based therapies may be capable of elucidating the molecular mechanisms of t-erbB2 forms in trastuzumab resistance and responsiveness to other therapeutics.

### Identify HER2 population in heterogeneous clinical samples

Tumor classification based on protein expression is used to stratify HER2-positive breast cancer patients.^10,36^ New classifications including both p185HER2 and t-erbB2 protein forms (p95, p110 or Δ16) could further inform selection of treatment regimen, such as kinase inhibitors (e.g., lapatinib) instead of trastuzumab alone.^17,33^ The scWB serves as an electrophoretic cytopathology tool, measuring both p185HER2 and t-erbB2 proteins with single-cell resolution. The assay aims to refine the HER2-positive breast cancer classification taxonomy by including t-erbB2 protein levels as a classifier. In a pilot study, eight primary breast tumor tissues were scrutinized by scWB, including four HER2-IHC 3+ patients, one HER2-IHC 2+ patient, and two HER2-IHC negative (0 or 1+) patients with T1–T4 tumor stage, with either positive or negative lymph node status. Available clinicopathologic details are in Supplementary Table 1.

**Fig. 3:**
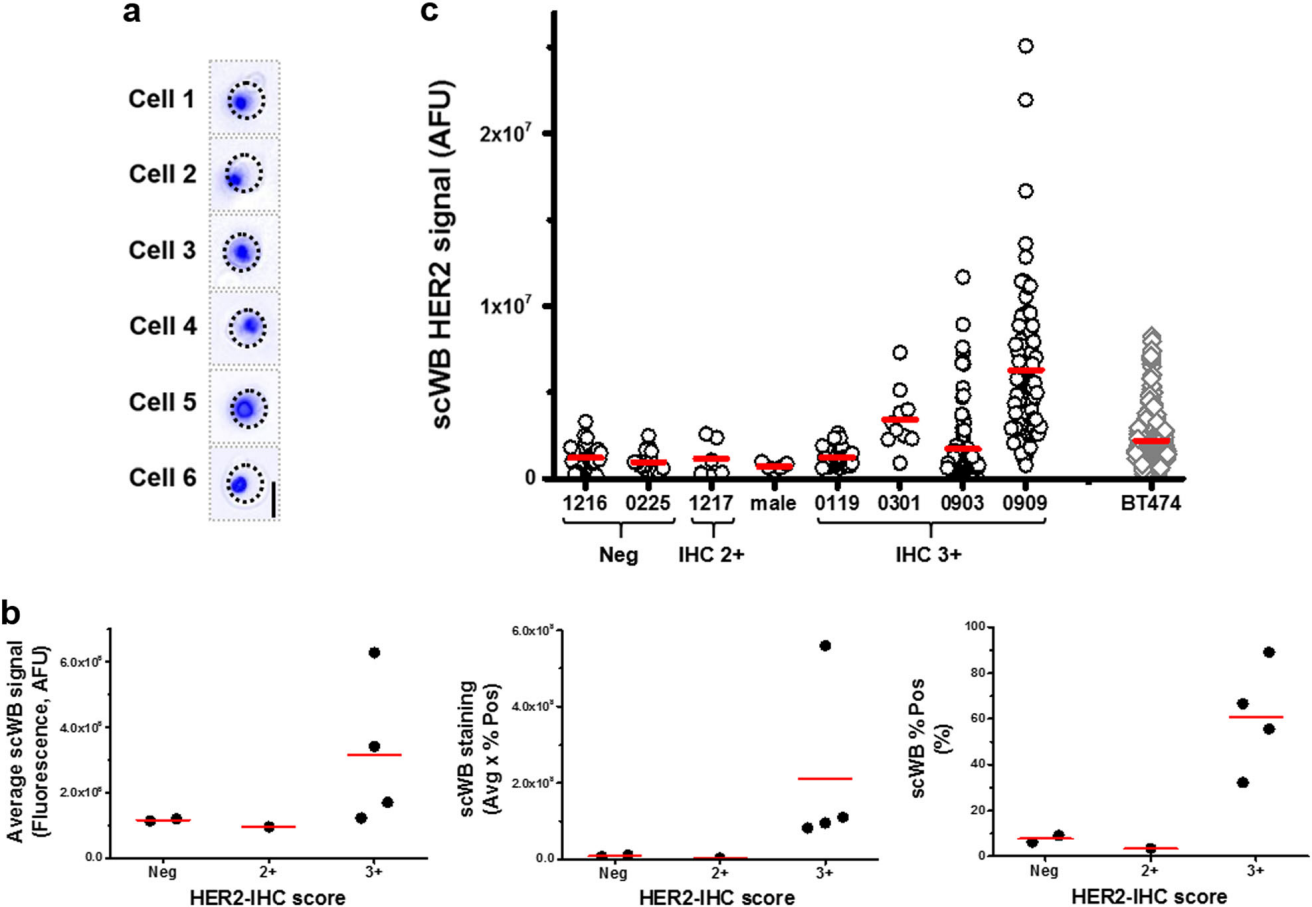
HER2-positive populations identified by scWB among primary breast tumor biopsies is in agreement with HER2-IHC score. **a** Overlaid bright field and fluorescence false-color micrographs of single primary breast cancer cells seated in microwells. The primary cells were stained by Hoechst 33342 (in blue). Microwell perimeter is marked by dashed lines. The scale bar is 30 μm. **b** The dot plots of histological HER2-IHC score with average scWB signal (AVG_HER2_, left panel), percentage of HER2-positive cells (% Pos, middle panel), and scWB staining (AVG_HER2_ multiplies by %Pos, right panel) show relative agreement among these breast tumor biopsies. **c** p185HER2 expression in individual cells from eight patient-derived breast tumor biopsies quantified by scWB show that among HER2-IHC 3+ tumors (0119, 0301, 0909, 0903), the p185HER2 expression distributions differ significantly (comparing Tumor 0119 and Tumor 0909, Mann-Whitney *u*-test, two-tailed, *p* < 1 × 10^−5^, *n*_t0119_ = 36 cells, *n*_t0909_ = 90 cells)

After tumor dissociation, single primary cells were settled into microwells for scWB analyses (Fig. 3a and Supplementary Table 2). Owing to the low cell density and the use of gravity-driven cell settling (sedimentation), the cell settling efficiency was 7% and single-cell microwell occupancy was 11% for Tumor 0903. We assayed six protein markers by the scWB via three immunoprobing rounds to identify the HER2-positive population. First, actinin and GAPDH differentiated cells from cell debris or empty microwells. Second, panCK distinguished epithelial cells from other contaminate cells (fibroblasts, macrophages, or endothelial cells). Third, the HER2-3B5 antibody identified HER2-positive breast cancer cells. Finally, the ratio of rs6 and p-rs6 implicated PI3K/AKT/mTOR signal activation. HER2 protein expression per cell (area-under-curve, AUC_HER2_) by scWB agreed with the HER2-IHC score determined by pathologists (Spearman’s ρ = 0.84, *p* = 0.02, *n* = 7 patients) (Fig. 3b). A similar trend was observed using the percentage of HER2-positive cells (%Pos) and the scWB staining intensity (average HER2 expression per tumor multiplied by %Pos). The observation of low HER2 protein expression quantified by the scWB in the HER2-IHC 2+ tumor was in agreement with the corresponding FISH classification (negative; HER2:CEP17 = 1.64 < 2.0,^37^ Supplementary Table 1). The discordance between IHC and FISH readouts can originate from tumor heterogeneity, sampling bias, and even subjective interpretation of IHC. Notably, we observed a high percentage of HER2-positive breast cancer cells (30–90%Pos) in HER2-IHC 3+ tumors, with only 6 and 9%Pos results in HER2-IHC 0–1+ tumors (Fig. 3b). This finding corroborates clinical HER2 classification: uniform, intense HER2 membrane staining of >30% of tumor cells justifies a HER2-IHC staining classification of 3+.^20^

Next, we examined the tumor-to-tumor variation of full-length HER2 (p185HER2) expression among all breast tumors considered here (Fig. 3c). We found no notable difference of p185HER2 expression in the breast tumor from a male patient compared to female-derived tumors (Fig. 3c and Supplementary Fig. 5). Among all HER2-IHC 3+ breast tumors (Tumor 0119, 0301, 0903, 0909), we found a 5-fold difference in p185HER2 expression (AVG_HER2, t0909_ = 6.3 ± 4.1 × 10^6^ (s.d.), *n* = 90 HER2-positive cells; AVG_HER2, t0119_ = 1.2 ± 0.54 × 10^6^ (s.d.), *n* = 36 HER2-positive cells). Within tumors, we observed a wide distribution of HER2 expression by scWB within Tumor 0909 (32-fold difference between the highest and lowest HER2 signal). When comparing HER2-IHC 3+ breast tumors with the HER2-IHC 3+ BT474 cell line,^38^ we observed that HER2 expression had higher variance in clinical Tumor 0909 and Tumor 0903 biopsies by scWB (*F*-test, *F*_t0909-BT474 = _8.41 > *F*_89,390_(0.05) = 1.44, *p* < 0.01; *F*_t0903-BT474 = _1.8 > *F*_126,390_(0.05) = 1.38, *p* < 0.01). As the HER2 heterogeneity observed by scWB in this first cohort of clinical breast tumor samples exceeds our previously established scWB intra-assay technical variation threshold of 32% CV,^39^ we attribute the measured HER2 variation to biological differences between cells (CV_t0909_ = 65%; CV_t0903_ = 111%; CV_t0119_ = 44%; CV_t0301_ = 52%). Tumor-to-tumor variation in HER2 expression may underlie differences in therapeutic response of HER2-IHC 3+ breast cancer patients after a single-agent trastuzumab treatment.

### Multiplexed scWB analyzes single t-erbB2-expressing cells

**Fig. 4:**
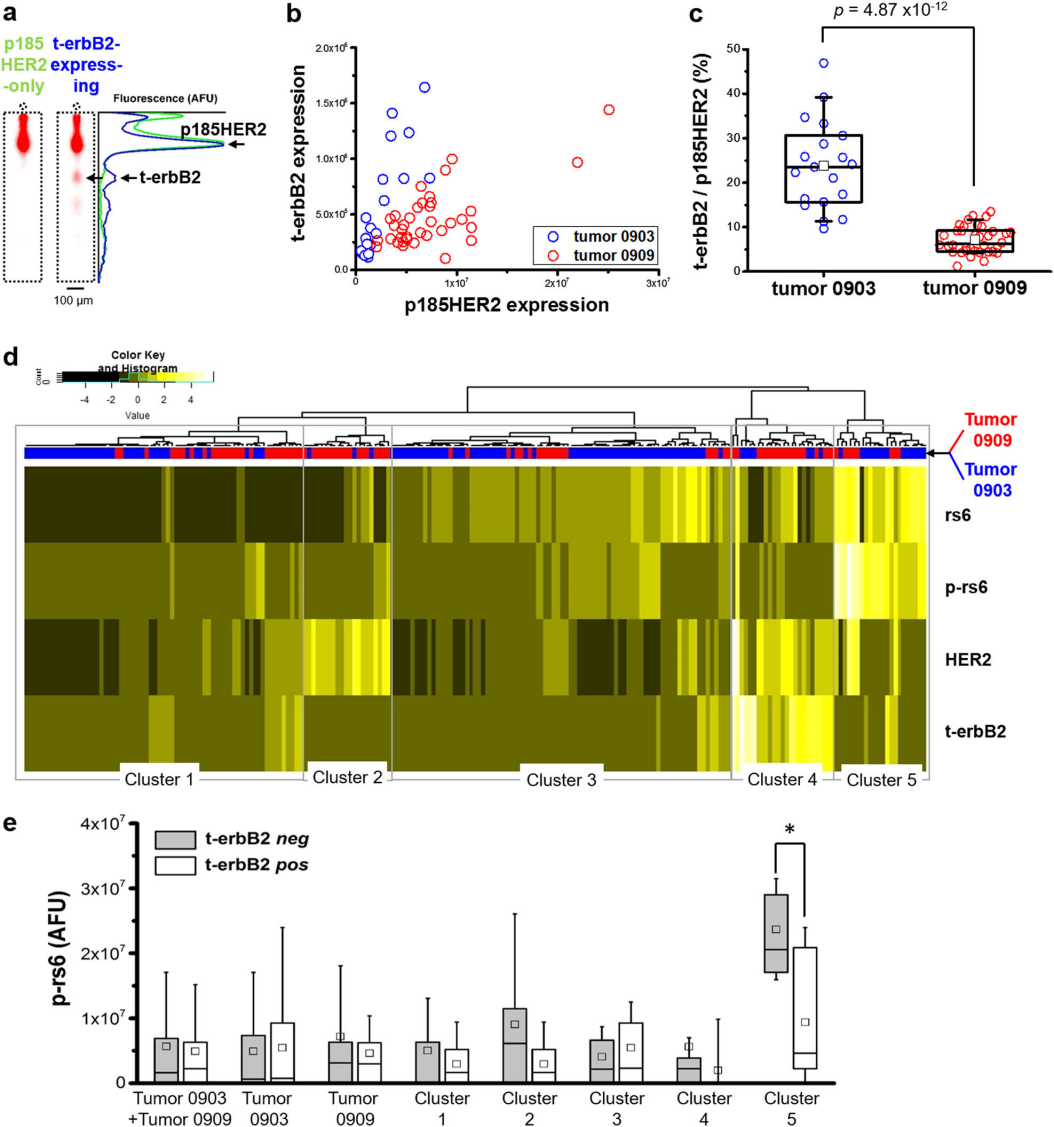
Hierarchical clustering reveals a p-rs6 activated subpopulation of cells from primary HER2-positive breast tumor biopsy specimens. **a** False-color fluorescence micrographs and intensity plots show a selected pair of t-erbB2-expressing and p185HER2-only primary breast tumor cells from Tumor 0909. The electrophoretic separation and immunoprobing by HER2-3B5 antibody identifies t-erbB2-expressing cells in primary tumor biopsies. **b** The scatter plot shows the expression of t-erbB2s and p185HER2 in tumor 0903 and 0909. **c** The box plot of t-erbB2s to p185HER2 expression ratio shows a statistically significant distribution difference between Tumor 0903 and 0909 (Mann-Whitney *u*-test, two-tailed, *p* < 0.01, *n*_t0903_ = 127 cells, *n*_t0909_ = 90 cells). **d** The heatmap shows characterization of cell populations from Tumor 0909 (red bar) and Tumor 0903 (blue bar) and scaled expression of target proteins (rs6, p-rs6, p185HER2, t-erbB2 in rows). Heat intensity reflects the standardized expression of each protein with white indicating highest expression and black indicating least expression. Cell populations were arranged according to linkage of hierarchical clustering. Five cell subpopulations were identified from clustering. The dendrograms were created using the Euclidean distance between expression of four scaled protein markers in each cell. **e** The box plots show p-rs6 expression between t-erbB2 negative (*neg, gray*) and positive (*pos, white*) populations in combined (Tumor 0909 and Tumor 0903), separated (Tumor 0909 or Tumor 0903), or clustered (cluster 1–5) samples. The p-rs6 expression is statistically different between t-erbB2s negative and positive populations in cluster 5 (Mann-Whitney’s *u*-test, two-tailed, *p* < 0.01, *n*_t-erbB2-*pos*-cluster 5_ = 11 cells, *n*_t-erbB2-*neg*-cluster 5_ = 11 cells, labeled as *). Box ends indicate 25th and 75th quantiles; black line at box indicates median value; black square at box indicates mean value; whiskers extend to 90% confidence limits. AFU Arbitrary fluorescence unit

scWB analysis of two tumors (Tumor 0903 and Tumor 0909) reported t-erbB2-expressing primary cells. Tumor 0903 exhibited 15% (19/127) and Tumor 0909 exhibited 40% (36/90) of the total HER2-positive breast cancer cells as expressing the t-erbB2 protein forms. Similar to analyses of the BT474 cell lines, we observed the co-existence of t-erbB2 isoforms/CTFs and the full-length p185HER2 protein in the same breast tumor cells (Fig. 4a). Intriguingly, while all t-erbB2 protein forms found in the BT474 cell lines electromigrated more slowly than actinin, some t-erbB2 forms electromigrated faster than actinin when derived from the clinical tumor cells (Supplementary Fig. 3). In the two tumors considered, the estimated molecular masses of the t-erbB2 proteins are 89.2 ± 10.5 kDa (*n*_t0903_ = 3 cells) and 124.0 ± 15.0 kDa (*n*_t0903_ = 11 cells), from the Ferguson plot generated using primary cells (Supplementary Fig. 6). Further, the scatter plot (Fig. 4b) shows appreciable correlation between t-erbB2s and p185HER2 expression (Spearman’s *ρ*_t0903_ = 0.84, *n*_t0903_ = 19, *p* = 7 × 10^-6^; Spearman’s *ρ*_t0909_ = 0.48, *n*_t0909_ = 36, *p* = 0.003). The measured correlation is consistent with the positive correlation between t-erbB2s expression and HER2-IHC score observed by other groups,^32,40^ supporting the hypothesis that at least a fraction of these truncated HER2 proteins may originate from protease shedding of full-length p185HER2 protein.

In comparing Tumor 0903 and Tumor 0909, we observed no statistically significant difference with regards to t-erbB2s expression (Mann-Whitney *u*-test, two-tailed, *p* = 0.99, *n*_t0903_ = 19, *n*_t0909_ = 36). Both tumors have ~3–4-fold changes in t-erbB2s expression (Fold change_t0903_ = 3.23, Fold change_t0909_ = 3.86). Intriguingly, we observed a statistically significant difference of t-erbB2s to p185HER protein expression ratio in Tumor 0903, comparing to that of Tumor 0909 (Fig. 4c; Mann-Whitney *u*-test, two-tailed, *p* = 4.87 × 10^−12^). Recent reports suggest that the high t-erbB2s to p185HER2 protein expression ratio significantly associates with shortened time to tumor progression (as indicated by persistence on protocol) of metastatic breast cancer patients compared to a high p185HER2 to t-erbB2s protein expression ratio; however, the patient exhibiting a high t-erbB2 to p185HER2 protein ratio benefitted from lapatinib treatment with a persistence on protocol of 17 months.^18^ In this pilot study, primary cells were acquired from a first tumor resection, consequently no trastuzumab-treatment results are available for these t-erbB2-expressing cells (i.e., Tumor 0903 and Tumor 0909) or p185HER2-only expressing cells (i.e., Tumor 0301 and Tumor 0119).

Given the multiplexing capability of the scWB, we next analyzed the association between t-erbB2s expression and PI3K/AKT/mTOR signal activation through rs6 phosphorylation. Fig. 4d shows the hierarchical clustering of cells in both tumor 0903 and 0909 based on HER2, t-erbB2s, rs6, and p-rs6. The dendrograms use the Euclidean distance between the scaled expression of four protein markers in each cell.^41^ From the hierarchical clustering, we visually identified 5 subpopulations (cluster 1–5) in the dendrogram. The presence of 5 clusters/subpopulations was also suggested by the eigenvalue distribution resulting from a spectral clustering analysis on the same data set. Intriguingly, while cluster 1 comprised cells almost evenly from Tumor 0903 and Tumor 0909, we found cluster 2 and 4 comprised cells primarily from Tumor 0909 (85% and 72%, respectively). In contrast, cluster 3 and 5 comprised cells primarily from Tumor 0903 (77% and 64%, respectively). These results suggest that certain cell sub-populations from Tumor 0903 and Tumor 0909 share common “proteotypes”, while other cell sub-populations are unique to one tumor or the other.

Within the five clusters, while cells in cluster 1 have low expression of all markers, we observed cells in cluster 4 express high p185HER2 and t-erbB2s but low rs6 and p-rs6 (Fig. 4d and Supplementary Fig. 7). In contrast, cells in cluster 5 express the opposite proteotype, which is low p185HER2 and t-erbB2s expression but high rs6 and p-rs6 expression. To scrutinize the signaling effect of t-erbB2s, we investigated the correlation between t-erbB2s and p-rs6 (rs6 activation). We observed no significant correlation between t-erbB2s and p-rs6 expression in a combined sample analysis with all cells included (Fig. 4e, Spearman’s *ρ*_t-erbB2-p-rs6 (t0909+t0903)_ = −0.001, *p* = 0.99, *n*_t0909+t0903_ = 217 cells; Spearman’s *ρ*_t-erbB2-p-rs6 (t0903)_ = −0.02, *p* = 0.82, *n*_t0903_ = 127 cells; Spearman’s ρ_t-erbB2-p-rs6 (t0909)_ = −0.02, *p* = 0.82, *n*_t0909_ = 90 cells). When checking the expression correlation between t-erbB2s and p-rs6 within each cluster, we observed cluster 5 had a decreasing monotonic trend between t-erbB2s and p-rs6 (Fig. 4e and Supplementary Fig. 8, Spearman’s *ρ*_t-erbB2-p-rs6 (cluster 5)_ = −0.62, *p* = 0.002, *n*_cluster 5_ = 22 cells). While full-length HER2 protein does activate PI3K/AKT/mTOR signaling which results in rs6 phosphorylaion,^35,42^ this cluster-specific (or subpopulation-specific) correlation analysis revealed that other mechanisms might affect the PI3K/AKT/mTOR signaling in the t-erbB2-expressing populations. As a corollary question, we sought to understand if rs6 phosphorylation may be associated with specific t-erbB2 forms. As such, we segregated the t-erbB2s having a smaller molecular mass (<100 kDa, e.g., p95HER2 fragments) from t-erbB2s having a larger molecular mass (>100 kDa, e.g., p110 isoforms). Although the small sample size precludes formal statistical analyses, qualitatively we did observe higher p-rs6 and a higher p-rs6 to rs6 ratio in p110 isoform-expressing cells (Supplementary Fig. 9). These intriguing—yet inconclusive—results point towards further single-cell resolution study of PI3K/AKT/mTOR signaling and signal activation of t-erbB2 in clinical breast cancer biopsies.

## Discussion

Like other oncoproteins, HER2 receptor biology is complex. Classifications of HER2-positive tumors including both canonical and truncated protein forms could, in theory, aid in informing precise treatment stratification for patients. For example, HER2-IHC 3+ patients are often treated with trastuzumab with or without pertuzumab, a HER2-dimerization inhibiting humanized monoclonal antibody. However, patients with either high t-erbB2 expression or high t-erbB2s to p185HER2 protein ratio can benefit from tyrosine kinase inhibitor treatments (i.e., lapatinib), without conferring trastuzumab resistance.^17,18,33^ Inclusion of t-erbB2s into HER2 classification schemes and a more comprehensive understanding of the mechanism(s) of t-erbB2s generation could provide insight into breast cancer pathogenesis, HER2-positive breast patient classification, and treatment considerations. Thus, if future larger clinical validation studies confirm these pilot findings, we view the scWB as a potentially promising advancement in clinical HER2 classification. The scWB readily resolves t-erbB2s from p185HER2 at single-cell resolution—using just one antibody probe—and most importantly enables multiplexing (which we demonstrate is capable of relating downstream signal transduction events with patterns of t-erbB2 expression)^28^ and long-term archiving.^29^

Here, multiplexed and quantitative scWB identifies t-erbB2-expressing cell subpopulations from heterogeneous clinical samples. The scWB resolved full-length (p185HER2) from truncated protein forms which can be further validated through extraction of the protein peak and matrix-assisted laser desorption time-of-flight mass spectrometry (MALDI-TOF) or electrospray tandem mass spectrometry (ESI MS) peptide mass mapping.^43^ In these preliminary studies, the HER2 scWB agreed with companion histological classifications (HER2-IHC score), while simultaneously reporting truncated isoform/CTFs expression. PAGE and immunoprobing allow selective t-erbB2 protein forms measurements from clinical samples. Averaged scWB results revealed that the total t-erbB2s expression accounts for just 2–5% of total HER2 expression in clinical HER2-positive breast biopsies, corroborating the limited capability of conventional western blotting in identifying t-erbB2-expressing subpopulations. Minor—but important—cell subpopulations are often masked in pooled analyses of heterogeneous clinical samples, thus impeding advances in t-erbB2s mechanistic insights. In this pilot study, the scWB identified 25% (2/8) t-erbB2-expressing tissues among all HER2-positive tissues, similar to the percentage (26–30%) reported by other research groups.^4,12,31,32^ Moreover, as noted by others,^4^ t-erbB2-expressing tissues are also p185HER2-positive, and the scWB verified that t-erbB2s co-exist and positively correlate with p185HER2 in the same primary cell. Additionally, the scWB gave not only average but single-cell level t-erbB2 and p185HER2 expression information. Measuring the percentage of cells with high t-erbB2-expression, high t-erbB2 to p185HER2 expression ratio, or a combination of t-erbB2 with other resistance markers can bring deeper insights to patient classification for precision medicine. Further, screening of other anti-tumor drugs specifically on t-erbB2-expressing cell subpopulations can accelerate development of personalized medicine for patients with specific t-erbB2 expression profiles.

Besides identification of the t-erbB2-expressing population, the multiplexed scWB provides the first examination of the signaling transduction of t-erbB2-expressing cells by measuring signaling related protein markers (i.e., rs6 and p-rs6) from primary breast biopsies. Phosphorylated rs6 can be a post-treatment indicator of HER2-positive breast cancer.^35,42^ Cell cluster analysis revealed a subpopulation of t-erbB2-positive breast cancer cells (22 cells) having less rs6 activation than found in t-erbB2-negative subpopulations in the same cluster. Further, cells that were positive for the larger molecular mass t-erbB2 forms showed qualitatively greater rs6 activation than cells having the smaller molecular mass forms. Given interest in both the function of rs6 in trastuzumab treatment response and the role of specific t-erbB2 forms (i.e., p110 isoform) in treatment resistance, we are actively developing single-cell western blotting capable of resolving all t-erbB2 forms.

Looking beyond the HER2 oncoprotein, we are interested in scrutinizing the truncated estrogen receptor isoform (ERα36) in tumor progression and metastasis of triple negative breast cancer,^44^ the BAG-1 isoforms in tumorigenesis and chemoresistance,^45^ and a new Bruton’s tyrosine kinase isoform (p65BTK) found in colon cancer.^46^ While both alternative translation at internal ribosome entry sites, IRES^47,48^ and shedding/activation of oncoproteins^49,50^ have received increased attention in tumorigenesis, selective measurement tools are needed to advance understanding in these areas. By relaxing the immunoassay requirement of selective, isoform-specific antibody probes, the scWB could facilitate rapid validation of rare oncoprotein tumor markers with relevance to personalized treatment decisions.

## Methods

### SU8 mold and the scWB gel slide fabrication

The SU8 mold fabrication was performed using manufacturer’s instructions (MicroChem) and previous publications.^28,51^ For the CHO cell experiments, microwell diameters and heights were 20 and 30 μm, respectively. For the BT474 and primary HER2-positive breast cancer cell experiments, microwell diameters and heights were 30 μm and 40–45 μm respectively. The microwell-to-microwell distance (separation length) was 1-mm. The scWB gel slides were fabricated against the SU8 mold. The 7%T, 2.6%C PAG was chemically polymerized using 0.08% ammonium persulfate (APS) and 0.08% tetramethylethylenediamine (TEMED). The PAG fabrication uses our previously reported process.^51^

### Cell lines

The genetically engineered CHO cell lines and the BT474, a HER2-positive breast cancer cell line, were obtained from Dr. Mark Pegram’s laboratory and maintained in RPMI 1640 media (#11875-093, Thermofisher Scientific) supplemented with 1% penicillin/streptomycin (#15140122, Thermofisher Scientific) and 10% of fetal bovine serum (FBS; #100-106, Gemini Bio-Products) and maintained in a humidified 37 °C incubator with 5% CO_2_. The CHO cells were genetically engineered with p185HER2, p110HER2 and p95HER2. The cDNA sequences encoding p185HER2, p110HER2 and p95HER2 were cloned into the pLVX puro lentiviral vector (Clontech).^32^ For consistency in this paper, we named the CHO cells engineered with p95HER2 as CHO/t-erbB2 in Fig. 1b,c. The CHO/p185 and CHO/t-erbB2 lines were selected under 0.55 mg mL^−1^ Geneticin (#10131027, Thermofisher Scientific). The expression levels of these forms were assessed using conventional western blot (Supplementary Fig. 1). Importantly, parental CHO cells showed negligible endogenous expression of p185HER2 and lacked detectable t-erbB2, thus only expression of these isoforms was observed in these cells. For the scWB, the cells were harvested by 5 mM EDTA/PBS (#15575020, Thermofisher Scientific), pelleted by centrifugation, and resuspended in PBS. Three or four replicates were assayed for each cell line samples (BT474 or CHO cells). There are total 273 quantifiable CHO/t-erbB2 cells, 211 quantifiable CHO/p183HER2 cells, and 391 quantifiable BT474 cells for generating the results. The cell solution was filtered through a 35 μm nylon cell strainer (#352235, BD Bioscience) to create a single-cell suspension prior to cell settling. The BT474 and CHO parental cell lines were tested to be free from mycoplasma. The BT474 cells were authenticated using STR (Short Tandem Repeat) profiling method by UC Berkeley Cell Culture Facility.

### Clinical breast tumor biopsies

Eight primary HER2-positive tissues were procured from the Stanford Tissue Bank under Institutional Review Board (IRB) approved protocols. The tissues were dissociated into single cells by using general tissue digestion procedures and the details are described as previously.^30^ In brief, the tissues were dissociated into single cells using fresh collagenase III (3000 unit/ml, C0255, Sigma-Aldrich). After neutralization by RPMI 1640 media supplemented with 10% FBS, the tissue suspension was pelleted by centrifugation. The red blood cell lysis step was skipped to reduce the cell loss during the centrifugation step. The cell pellet was resuspended in 0.5 mL of FBS supplemented with 10% (vol/vol) DMSO and then aliquoted into 3–5 tubes, each with 100 µl cell suspension. The primary HER2-positive breast cancer cells were stored at −86 °C freezer before use.

In the scWB, the primary HER2-positive breast cancer cells were rapidly thawed and pelleted by centrifugation. The cells were resuspended in ice-cold HBSS (#14175095, Thermofisher Scientific) supplemented with 1% (vol/vol) protease/phosphatase inhibitor cocktail (#78446, Thermofisher Scientific) and settled on the scWB gel slide for 10 to 30 min before performing the scWB. We assayed 6 protein markers by the scWB via 3 immunoprobing rounds to identify the HER2-positive population in the heterogeneous breast tumor biopsies. The quantifiable HER2-positive cell number of each tumor is 90 cells for Tumor 0909, 127 cells for Tumor 0903, 36 cells for Tumor 0119, 10 cells for Tumor 0301, 42 cells for Tumor 1216, 6 cells for Tumor 1217, 15 cells for Tumor 0225, and four cells for the male breast tumor. The cell number of panCK-positive cells in each tumor is listed in the Supplementary Table 1.

### scWB protocol

The handling of scWB on cell lines and primary cells, including single-cell settling, in situ lysis, PAGE, photo-activated protein immobilization and immunoprobing, has been previously reported.^29,30,51^ Briefly, the cells were settled in 20 µm diameter microwells for CHO/p185 and CHO/t-erbB2 cells and 30 µm diameter microwells for BT474 and primary HER2-positive breast cancer cells. The cells were lysed in situ by 55 °C, 0.5% SDS lysis buffer for 20 s in CHO cells and 25 s in BT474 and primary HER2-positive breast cancer cells. For detecting phosphoproteins, 0.1% (vol/vol) protease/phosphatase inhibitor cocktail (#78446, Thermofisher Scientific) was added to the lysis buffer immediately before pouring. The single-cell protein lysates were all electromigrated in 7%T, 2.6%C PAG for 15 s with an applied electric field of 40 V cm^−1^. The separated proteins were immobilized to the benzophenone-incorporated gel by UV (100% power, Lightningcure, LC5, Hamamatsu) for 45 s. The immobilized proteins were then immunoprobed in-gel by primary and fluorescently-labeled secondary antibodies. A 4-laser fluorescence microarray scanner (Genepix 4300 A, Molecular Devices) was used to acquire fluorescence readout. For protein multiplexing, the stripping and antibody reprobing procedures have been detailed previously.^29,51^

### Imaging

Single primary cells were imaged on an Olympus IX71 inverted fluorescence microscope equipped with an Andor iXon+ EMCCD camera, ASI motorized stage, shuttered mercury lamp light source (X-cite, Lumen Dynamics), and controlled by MetaMorph software (Molecular Devices) at 1 × 1 pixel binning through a 10× magnification objective (Olympus UPlanFLN, NA 0.45). The fluorescence signal of Hoechst 33342 DNA stain (B2261, Sigma) was obtained using a standard DAPI filter cube. All images were analyzed by ImageJ 1.46r (NIH). All plots were graphed by OriginPro 8.5.0.

### Antibodies

The primary antibodies include GAPDH (goat pAb; SAB2500450, Sigma), βTub (rabbit pAb; ab6046, Abcam), α-actinin (rabbit mAb; 6487, Cell Signaling), HER2-3B5 (mouse mAb; ab16901, Abcam), HER2-2G11 (mouse mAb; AHO0918, Thermofisher Scientific), ERK1/2 (rabbit mAb; 4695, Cell Signaling), phospho-ERK1/2 (Thr202/Tyr204) (rabbit mAb; 4370, Cell Signaling), mTOR (rabbit mAb; 2983, Cell Signaling), panCK (rabbit pAb; Z0622, Dako), S6-ribosomal protein (mouse mAb; 2317, Cell Signaling), phospho-S6-ribosomal protein (Ser240/244) (rabbit mAb; 5364, Cell Signaling). The secondary antibodies include donkey anti-mouse IgG (H + L) (Alexa Fluor 488 conjugate, A21202, Thermofisher Scientific), donkey anti-goat IgG (H + L) (Alexa Fluor 555 conjugate, A21432, Thermofisher Scientific), and donkey anti-rabbit IgG (H + L) (Alexa Fluor 647 conjugate, A31573, Thermofisher Scientific).

### Data analysis and statistics

The protein quantification in the scWB experiments was performed using an in-house MATLAB script (Mathworks, Matlab R2016a). The guideline for scWB analysis can be found in the previously published protocol.^51^ Specifically, for separating p185HER2 and t-erbB2, we performed Gaussian curve fitting at selected boundary (p185HER2: 40–150 μm, t-erbB2: 150–300 μm). All proteins except t-erbB2 were quantified from area under curve (AUC) within ± 4 standard deviation based on Gaussian curve fitting with a SNR > 3 and *R*^2^ > 0.7 of background subtracted protein peaks. Owing to the proximity to p185HER2 peak, the t-erbB2-expressing cells were selected from the Gaussian curve fitting with a higher SNR (SNR > 10) owing to the residual signal from the tail of p185HER2 peak. Further, the t-erbB2 expression was quantified from AUC within a set boundary.

Multiple statistical analyses were performed. Spearman’s rank correlation coefficient was used to determine the correlation between the expressions of two proteins which was expected to be monotonic but not necessarily linear. The Mann-Whitney *u*-test was used to determine the difference in mean and distribution of two cell populations that were not necessarily normally distributed. The coefficient of variation (CV) or variance was employed to evaluate the cell-to-cell or tumor-to-tumor variation. The *F*-test was used to determine the equality of variances of expressions in two cell populations. Single-cell clustering was used to identify cell subpopulations within HER2-positive breast tumors (Tumor 0903 and Tumor 0909). The quantified protein expressions (AUC) were standardized for each protein using the “scale” method in R (version 3.3.2). Since the expression levels of the four proteins have been scaled and there is a small number of proteins, it is reasonable to use the Euclidian distance to assess the relationships between cells. Correlation-based measures may be less appropriate here because of the potential large variance of correlation estimates between vectors with very low dimensions. The dendrogram and heatmap were generated with R (version 3.3.2), using hierarchical clustering with the Euclidean distance function and the ‘ward.D2’ method. Normalized spectral clustering was performed by using an epsilon-neighborhood graph to construct an adjacency matrix with which the Laplacian matrix was computed.^52^ Eigenvalues resulting from the normalized eigenproblem involving this Laplacian matrix were plotted in order to determine the number of clusters (Supplementary Fig. 10).

### Data availability statement

The datasets produced during the current study have been deposited in the Figshare repository with the DOI: 10.6084/m9.figshare.c.4002240 (https://figshare.com/s/fc77c1ca77e6976f3c59).

## Electronic supplementary material

Supplementary Information

## References

[1] Arribas J, Baselga J, Pedersen K, Parra-Palau JL (2011). p95HER2 and breast cancer. Cancer Res..

[2] Yang CPH, Yap EH, Xiao H, Fiser A, Horwitz SB (2016). 2-(m-Azidobenzoyl)taxol binds differentially to distinct β-tubulin isotypes. Proc. Natl Acad. Sci. USA.

[3] Harper SJ, Bates DO (2008). VEGF-A splicing: the key to anti-angiogenic therapeutics?. Nat. Rev. Cancer.

[4] Christianson TA (1998). NH2-terminally truncated HER-2/neu protein: relationship with shedding of the extracellular domain and with prognostic factors in breast cancer. Cancer Res..

[5] Anido J (2006). Biosynthesis of tumorigenic HER2 C-terminal fragments by alternative initiation of translation. EMBO J..

[6] Pedersen K (2009). A naturally occurring HER2 carboxy-terminal fragment promotes mammary tumor growth and metastasis. Mol. Cell. Biol..

[7] Jackson C, Browell D, Gautrey H, Tyson-Capper A (2013). Clinical significance of HER-2 splice variants in breast cancer progression and drug resistance. Int. J. Cell Biol..

[8] Slamon DJ (2001). Use of chemotherapy plus a monoclonal antibody against HER2 for metastatic breast cancer that overexpresses HER2. N. Engl. J. Med..

[9] Yarden Y, Sliwkowski MX (2001). Untangling the ErbB signalling network. Nat. Rev. Mol. Cell Biol..

[10] Arteaga CL (2012). Treatment of HER2-positive breast cancer: current status and future perspectives. Nat. Rev. Clin. Oncol..

[11] Guarneri V (2015). Prospective biomarker analysis of the randomized CHER-LOB study evaluating the dual Anti-HER2 treatment with trastuzumab and lapatinib plus chemotherapy as neoadjuvant therapy for HER2-positive breast cancer. Oncologist.

[12] Sáez R (2006). p95HER-2 predicts worse outcome in patients with HER-2-positive breast cancer. Clin. Cancer Res..

[13] Sperinde J (2010). Quantitation of p95HER2 in paraffin sections by using a p95-specific antibody and correlation with outcome in a cohort of trastuzumab-treated breast cancer patients. Clin. Cancer Res..

[14] Tural D, Akar E, Mutlu H, Kilickap S (2014). P95 HER2 fragments and breast cancer outcome. Expert Rev. Anticancer Ther..

[15] Duchnowska R (2015). Quantitative HER2 and p95HER2 levels in primary breast cancers and matched brain metastases. Neuro. Oncol..

[16] Molina MA (2002). NH2-terminal truncated HER-2 protein but not full-length receptor is associated with nodal metastasis in human breast cancer. Clin. Cancer Res..

[17] Scaltriti M (2010). Clinical benefit of lapatinib-based therapy in patients with human epidermal growth factor receptor 2-positive breast tumors coexpressing the truncated p95HER2 receptor. Clin. Cancer Res..

[18] Montemurro F (2014). Potential biomarkers of long-term benefit from single-agent trastuzumab or lapatinib in HER2-positive metastatic breast cancer. Mol. Oncol..

[19] Kulasingam, V., Prassas I. & Diamandis, E. P. Towards personalized tumor markers. *npj Precis. Oncol*. 10.1038/s41698-017-0021-2 (2017). 10.1038/s41698-017-0021-2 PMC5871887 29872704

[20] Wolff AC (2013). Recommendations for human epidermal growth factor receptor 2 testing in breast cancer: American Society of Clinical Oncology/College of American Pathologists clinical practice guideline update. J. Clin. Oncol..

[21] Dupouy DG (2016). Continuous quantification of HER2 expression by microfluidic precision immunofluorescence estimates HER2 gene amplification in breast cancer. Sci. Rep..

[22] Djuric, U., Zadeh, G., Aldape, K. & Diamandis, P. Precision histology: how deep learning is poised to revitalize histomorphology for personalized cancer care. *npj Precis. Oncol*. 10.1038/s41698-017-0022-1 (2017). 10.1038/s41698-017-0022-1 PMC5871847 29872706

[23] Zhang L (2017). Light scattering spectroscopy identifies the malignant potential of pancreatic cysts during endoscopy. Nat. Biomed. Eng..

[24] Janiszewska M (2015). In situ single-cell analysis identifies heterogeneity for PIK3CA mutation and HER2 amplification in HER2-positive breast cancer. Nat. Genet..

[25] Kim KT (2015). Single-cell mRNA sequencing identifies subclonal heterogeneity in anti-cancer drug responses of lung adenocarcinoma cells. Genome Biol..

[26] Giesen C (2014). Highly multiplexed imaging of tumor tissues with subcellular resolution by mass cytometry. Nat. Methods.

[27] Leuchowius KJ (2013). Parallel visualization of multiple protein complexes in individual cells in tumor tissue. Mol. Cell. Proteom..

[28] Hughes AJ (2014). Single-cell western blotting. Nat. Methods.

[29] Kang CC, Lin JMG, Xu Z, Kumar S, Herr AE (2014). Single-cell western blotting after whole-cell imaging to assess cancer chemotherapeutic response. Anal. Chem..

[30] Duncombe TA (2015). Hydrogel pore-size modulation for enhanced single-cell western blotting. Adv. Mater..

[31] Molina MA (2001). Trastuzumab (herceptin), a humanized anti-Her2 receptor monoclonal antibody, inhibits basal and activated Her2 ectodomain cleavage in breast cancer cells. Cancer Res..

[32] Ward TM (2013). Truncated p110 ERBB2 induces mammary epithelial cell migration, invasion and orthotopic xenograft formation, and is associated with loss of phosphorylated STAT5. Oncogene.

[33] Scaltriti M (2007). Expression of p95HER2, a truncated form of the HER2 receptor, and response to anti-HER2 therapies in breast cancer. J. Natl Cancer Inst..

[34] Shi Y (2009). A novel proximity assay for the detection of proteins and protein complexes: quantitation of HER1 and HER2 total protein expression and homodimerization in formalin-fixed, paraffin-embedded cell lines and breast cancer tissue. Diagn. Mol. Pathol..

[35] Jegg AM (2012). PI3K independent activation of mTORC1 as a target in lapatinib-resistant ERBB2+breast cancer cells. Breast Cancer Res. Treat..

[36] Slamon DJ (1987). Human breast cancer: correlation of relapse and survival with amplification of the HER-2/neu oncogene. Science.

[37] Kaufman PA (2014). Assessing the discordance rate between local and central HER2 testing in women with locally determined HER2-negative breast cancer. Cancer.

[38] Subik K (2010). The Expression patterns of ER, PR, HER2, CK5/6, EGFR, Ki-67 and AR by immunohistochemical analysis in breast cancer cell lines. Breast Cancer.

[39] Sinkala E (2017). Profiling protein expression in circulating tumour cells using microfluidic western blotting. Nat. Commun..

[40] Lee J (2013). A novel proteomics-based clinical diagnostics technology identifies heterogeneity in activated signaling pathways in gastric cancers. PLoS ONE.

[41] Gebauer N (2014). Genomic landscape of pancreatic neuroendocrine tumors. World J. Gastroenterol..

[42] Yang-Kolodji G, Mumenthaler SM, Mehta A, Ji L, Tripathy D (2015). Phosphorylated ribosomal S6 (p-rpS6) as a post-treatment indicator of HER2 signalling targeted drug resistance. Biomarkers.

[43] Lim H (2003). Identification of 2D-gel proteins: a comparison of MALDI/TOF peptide mass mapping to μ LC-ESI tandem mass spectrometry. J. Am. Soc. Mass Spectrom..

[44] Chaudhri Ra, Hadadi A, Lobachev KS, Schwartz Z, Boyan BD (2014). Estrogen receptor-alpha 36 mediates the anti-apoptotic effect of estradiol in triple negative breast cancer cells via a membrane-associated mechanism. Biochim. Biophys. Acta.

[45] Dobbyn HC (2008). Regulation of BAG-1 IRES-mediated translation following chemotoxic stress. Oncogene.

[46] Grassilli, E. et al. A novel oncogenic BTK isoform is overexpressed in colon cancers and required for RAS-mediated transformation. *Oncogene.*10.1038/onc.2015.504 (2016). 10.1038/onc.2015.504 PMC4994017 26804170

[47] Faye MD, Holcik M (2015). The role of IRES trans-acting factors in carcinogenesis. Biochim. Biophys. Acta - Gene Regul. Mech..

[48] Komar AA, Hatzoglou M (2011). Cellular IRES-mediated translation: the war of ITAFs in pathophysiological states. Cell Cycle.

[49] Vidlickova I (2016). Apoptosis-induced ectodomain shedding of hypoxia-regulated carbonic anhydrase IX from tumor cells: a double-edged response to chemotherapy. BMC Cancer.

[50] Kyula JN (2010). Chemotherapy-induced activation of ADAM-17: a novel mechanism of drug resistance in colorectal cancer. Clin. Cancer Res..

[51] Kang CC (2016). Single cell-resolution western blotting. Nat. Protoc..

[52] Ng, A. Y., Jordan, M. I. & Weiss, A. Y. On spectral clustering: analysis and an algorithm. *Adv. Neural Inf. Process. Syst*. **14**, 849 (2002).

