# Supplementary material for: Electrophoretic cytopathology resolves ERBB2 forms with single-cell resolution
Source: NPJ Precis Oncol. 2018 Mar 22;2:10. doi: 10.1038/s41698-018-0052-3 (PMC5871910; doi:10.1038/s41698-018-0052-3)
Supplement: Supplementary file 1 — Supplementary Information [file 41698_2018_52_MOESM1_ESM.pdf]

Supporting information

Electrophoretic cytopathology resolves ERBB2 forms with single-cell resolution

Chi-Chih Kang[1], Toby M. Ward[2], Jessica Bockhorn[2], Courtney Schiffman[3], Haiyan Huang[4], Mark D. Pegram[2] and Amy E. Herr[1,*]

[1]Department of Bioengineering, University of California, Berkeley, CA 94720, USA

[2]Division of Oncology, Department of Medicine, Stanford University, Stanford, CA 94305, USA

[3]Division of Biostatistics, School of Public Health, University of California, Berkeley, CA 94720, USA

[4]Department of Statistics, University of California, Berkeley, CA 94720, USA

Table of Content

Supplementary Figure 1. The conventional western blot displayed p185HER2, p110HER2, and p95HER2 from different genetically engineered HER2 isoform CHO lines.

Supplementary Figure 2. The Ferguson plot was generated from proteins with known molecular masses.

Supplementary Figure 3. Electromigration of t-erbB2 comparing to actinin between BT474 and clinical breast tumor cells.

Supplementary Figure 4. Positive correlations exhibited between internal control proteins in BT474 cells.

Supplementary Figure 5. Similar HER2 expression micrographs exhibited in HER2-positive breast tumor biopsies from male and female patients.

Supplementary Figure 6. The Ferguson plot was established from proteins with known molecular masses from clinical samples.

Supplementary Figure 7. Cluster 4 exhibited high p185HER2 and t-erbB2 while cluster 5 exhibited high rs6 and p-rs6.

Supplementary Figure 8. The t-erbB2 and p-rs6 protein had monotonic decreasing relationship in the cluster 5.

Supplementary Figure 9. The t-erbB2 subpopulation analysis suggests primary breast cancer cells with larger t-erbB2 (>100 kDa) have rs6 activation comparing to cells with smaller t-erbB2 (<100 kDa).

Supplementary Figure 10. Spectral clustering confirms five clusters identified in the hierarchical clustering dendrogram.

Supplementary Table 1. Clinical-pathological characteristics of breast tumor biopsies.

Supplementary Table 2. Analysis of primary cells from tumor 0903 in the microwells.

Supplementary Figure 1.

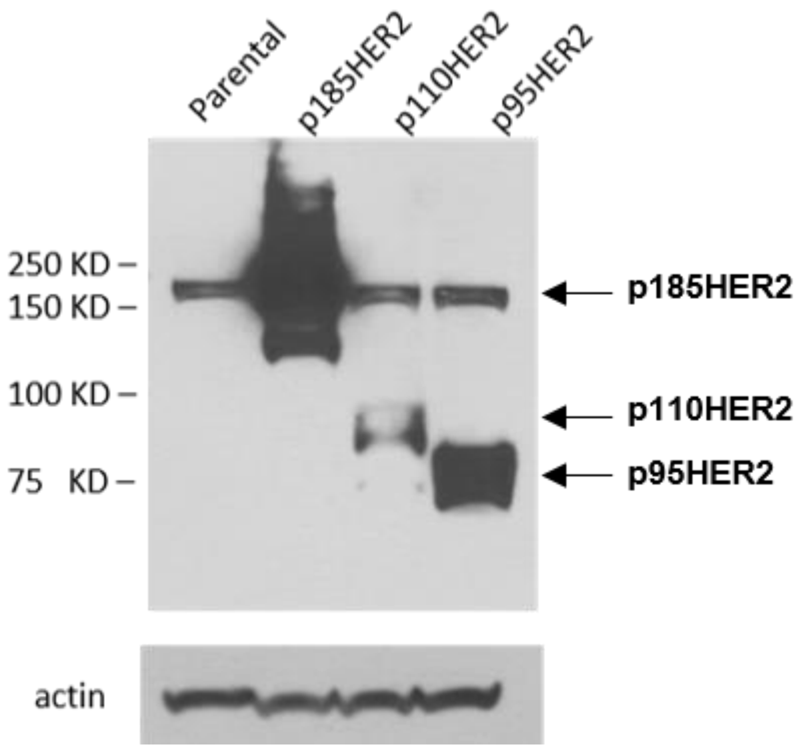

Supplementary Figure 1. The conventional western blot displayed p185HER2, p110HER2, and p95HER2 from different genetically engineered HER2 isoform CHO lines. The blotting result shows that genetically engineered p95, p110, and p185 HER2 protein isoforms electromigrated at different locations in a polyacrylamide gel. The Actin protein is shown here for a loading and location control.

Supplementary Figure 2

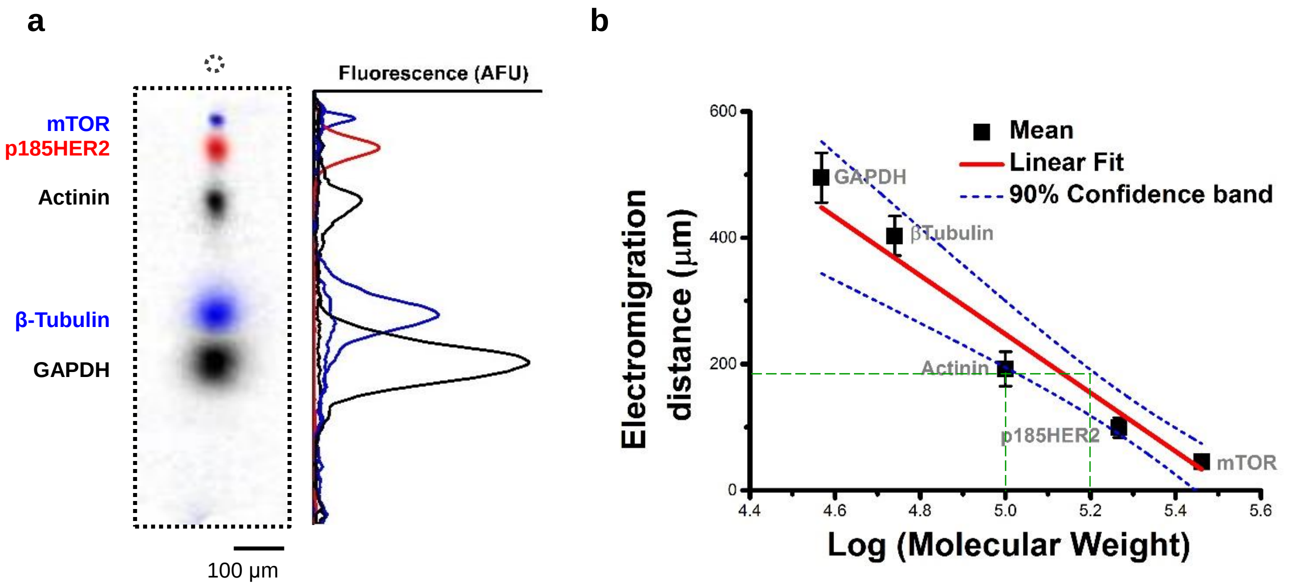

Supplementary Figure 2. The Ferguson plot was generated from proteins with known molecular masses. (a) False-color fluorescence micrographs and intensity plots show the electromigration of different proteins (mTOR:289 kDa: blue, p185HER2:185 kDa: red, Actinin:100 kDa: black, β-Tubulin:55 kDa: blue, and GAPDH:37kDa: black) with known molecular masses in 7%T, 2.6%C PAG. The electromigration distance of each protein was quantified by Gaussian fitting (L_{mTOR}: 44.7 ± 10.4 (s.d.) μm, n = 367 cells; $L_{p185HER2}$: 99.3 ± 15.7 (s.d.) μm, n = 375 cells; $L_{Actinin}$: 192.5 ± 27.2 (s.d.) μm, n = 374 cells; $L_{\beta\text{-Tubulin}}$: 403.2 ± 31.3 (s.d.) μm, n = 379 cells; and L_{GAPDH}: 495.2 ± 39.5 (s.d.) μm, n = 392 cells, from 4 independent experiments). (b) The Ferguson plot shows a linear relationship between the electromigration distance and the log value of protein molecular mass (y = - 464x + 2569.8, R^2 = 0.94). The linear correlation allows further calculation of the molecular mass of t-erbB2 based on its electromigration distance. Red straight line indicates linear fit; blue dash lines cover the 90% confidence band; green dash lines indicate the electromigration distance of t-erbB2 and its corresponding molecular masses. The analysis was performed using OriginPro (9.0.0 OriginLab).

Supplementary Figure 3.

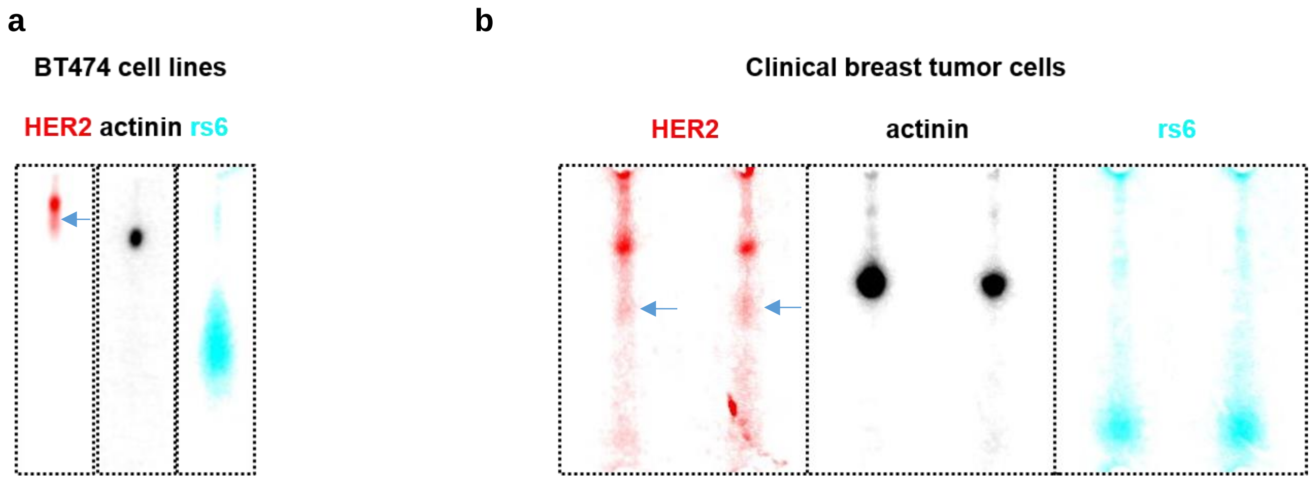

Supplementary Figure 3. Electromigration of t-erbB2 comparing to actinin between BT474 and clinical breast tumor cells. The fluorescence micrographs show the HER2 (red), t-erbB2 (indicated by arrow), actinin (black), and rs6 (cyan) in BT474 cells (a) and clinical breast tumor cells (b).

Supplementary Figure 4.

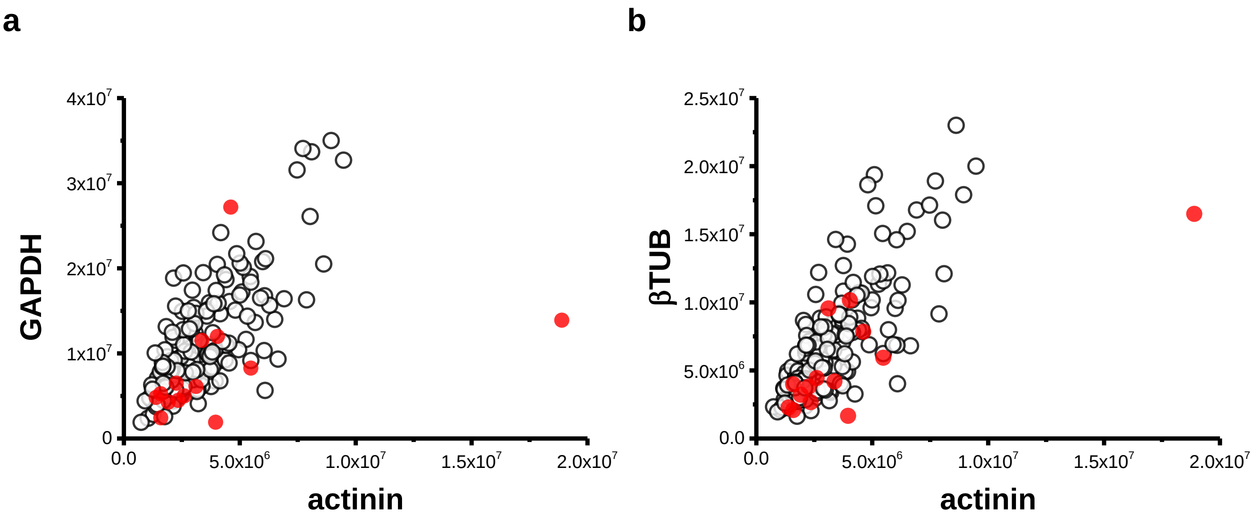

Supplementary Figure 4. Positive correlations exhibited between internal control proteins in BT474 cells. The scatter plots of actinin and GAPDH (a) and actinin and βTub (b) show monotonic relationship between internal control proteins (Spearman's $\rho_{actinin\text{-}GAPDH} = 0.7$, $p < 1 \times 10^{-6}$, n = 188 cells; Spearman's $\rho_{actinin\text{-}\beta Tub} = 0.76$, $p < 1 \times 10^{-6}$, n = 188 cells). Moreover, the expression ranges of GAPDH, actinin, and βTub in t-erbB2-expressing BT474 cells are within that in p185HER2-only BT474 cells. (◯) denotes p185HER2 only BT474 cells. (●) denotes t-erbB2-expressing BT474 cells.

Supplementary Figure 5.

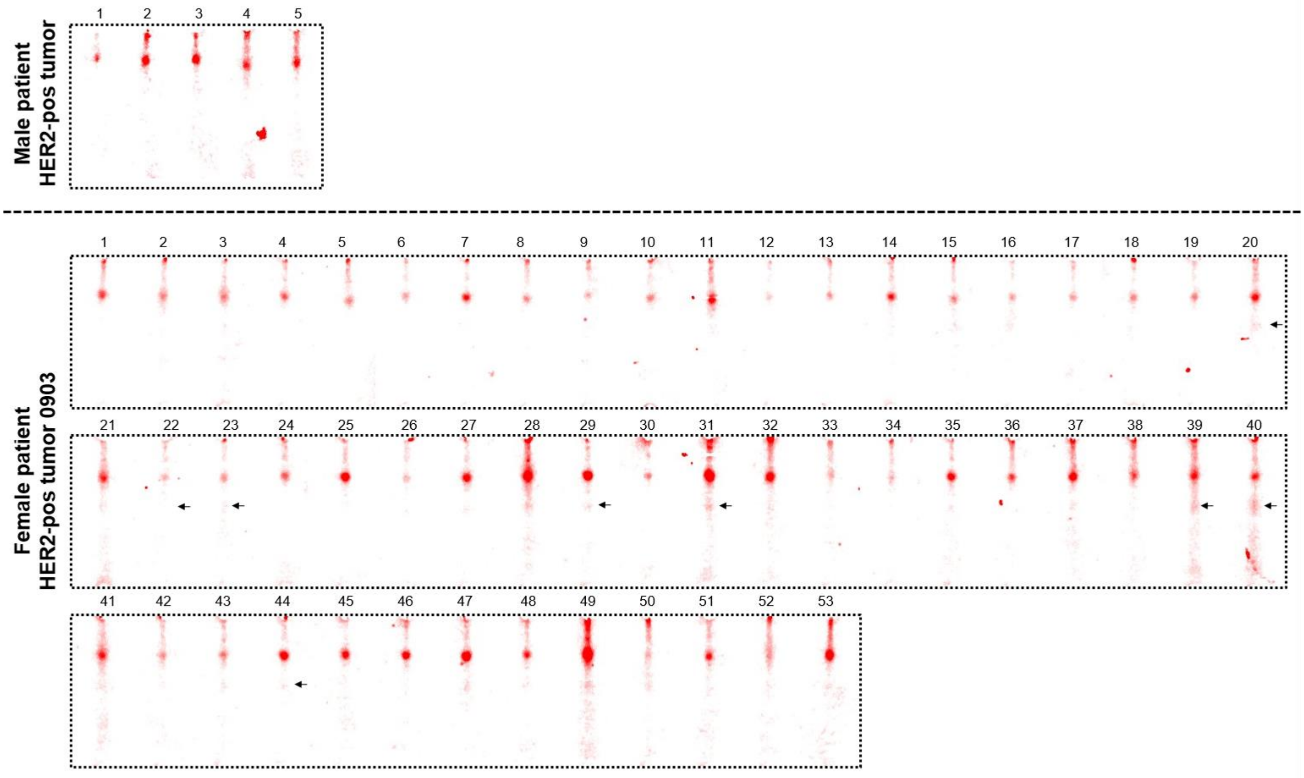

Supplementary Figure 5. Similar HER2 expression micrographs exhibited in HER2-positive breast tumor biopsies from male and female patients. Montage HER2 fluorescence micrographs from male patients (top panel) and female patients (bottom).

Supplementary Figure 6.

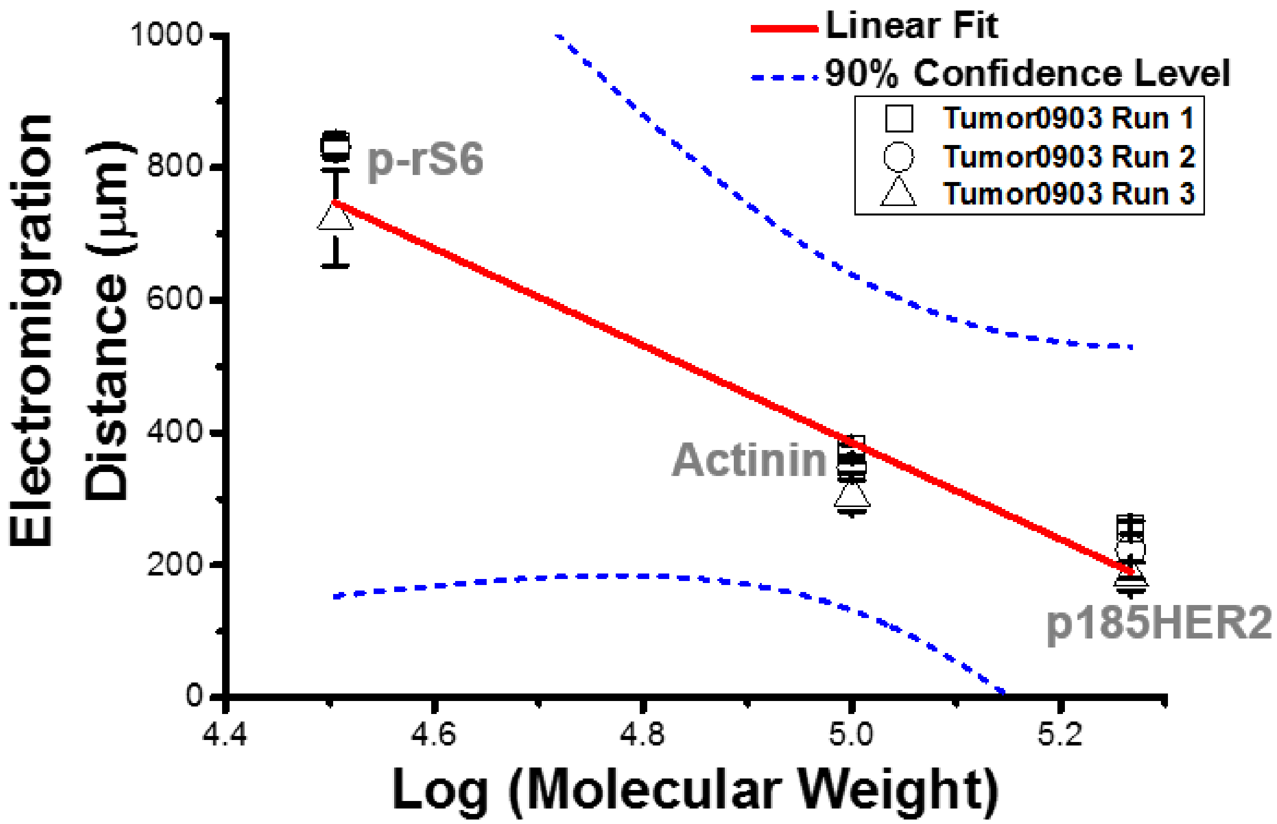

Supplementary Figure 6. The Ferguson plot was established from proteins with known molecular masses from clinical samples. The Ferguson plots shows a linear relationship between the electromigration and the log value of molecular masses from p185HER2, Actinin, and p-rS6 from Tumor0903. The linear relationship in Tumor0903 and is $y = - 731x + 4043$, $R^2 = 0.90$. Red straight line indicates linear fit; blue dash lines cover the 90% confidence band. The analysis was performed using OriginPro (9.0.0 OriginLab).

Supplementary Figure 7.

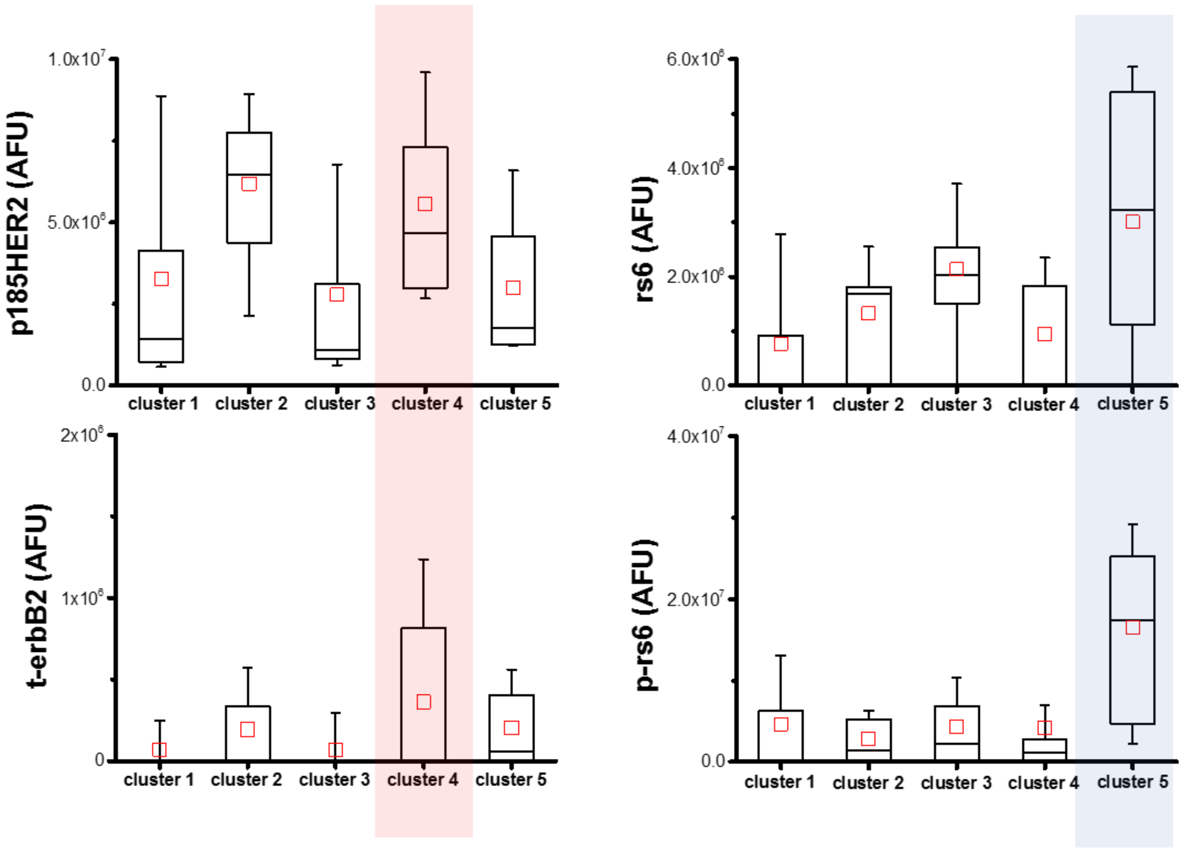

Supplementary Figure 7. Cluster 4 exhibited high p185HER2 and t-erbB2 while cluster 5 exhibited high rs6 and p-rs6. The box plots show the p185HER2, t-erbB2, rs6, and p-rs6 protein expression of cluster 1 to 5. Box ends indicate 25th and 75th quantiles; black line at box indicates median value; red square at box indicates mean value; whiskers extend to 90% confidence limits. AFU: Arbitrary fluorescence unit.

Supplementary Figure 8.

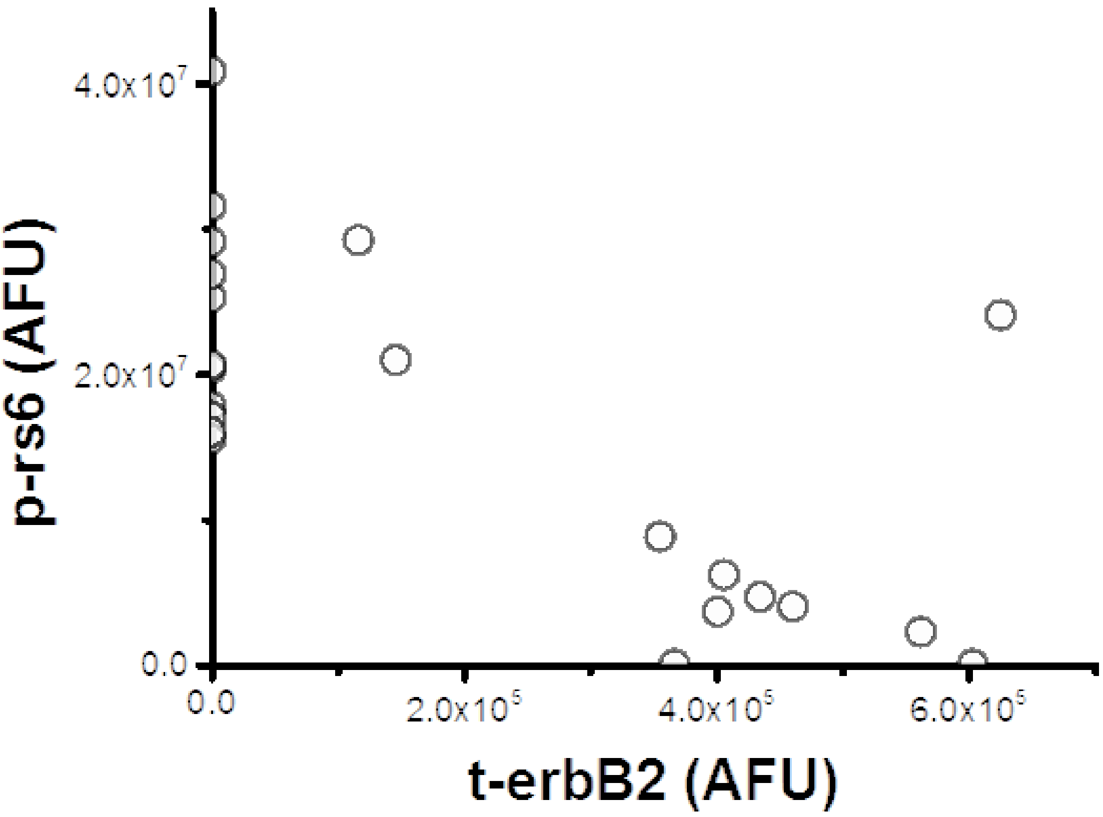

Supplementary Figure 8. The t-erbB2 and p-rs6 protein had monotonic decreasing relationship in the cluster 5. The scatter plot show the expression of t-erbB2 and p-rs6 in the cluster 5. AFU: Arbitrary fluorescence unit.

Supplementary Figure 9.

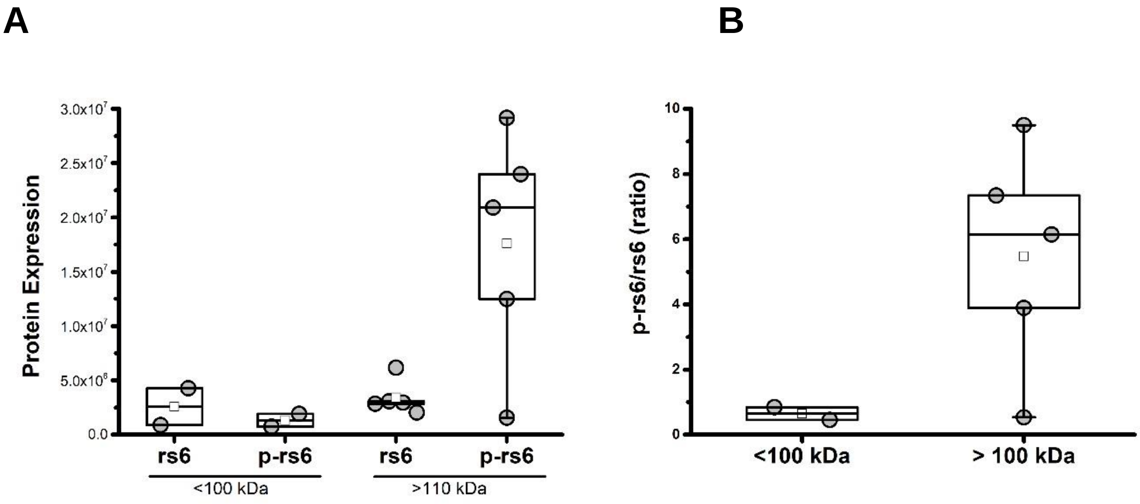

Supplementary Figure 9. The t-erbB2 subpopulation analysis suggests primary breast cancer cells with larger t-erbB2 (>100 kDa) have rs6 activation comparing to cells with smaller t-erbB2 (<100 kDa). The box plots show the rs6 and p-rs6 protein expression (A) and p-rs6 to rs6 ratio (B) of t-erbB2-expressing cells with larger t-erbB2 (> 100 kDa, n = 5 cells) and smaller t-erbB2 (<100 kDa, n = 2 cells). Box ends indicate 25th and 75th quantiles; black line at box indicates median value; the square at box indicates mean value; whiskers extend to 90% confidence limits. AFU: Arbitrary fluorescence unit.

Supplementary Figure 10.

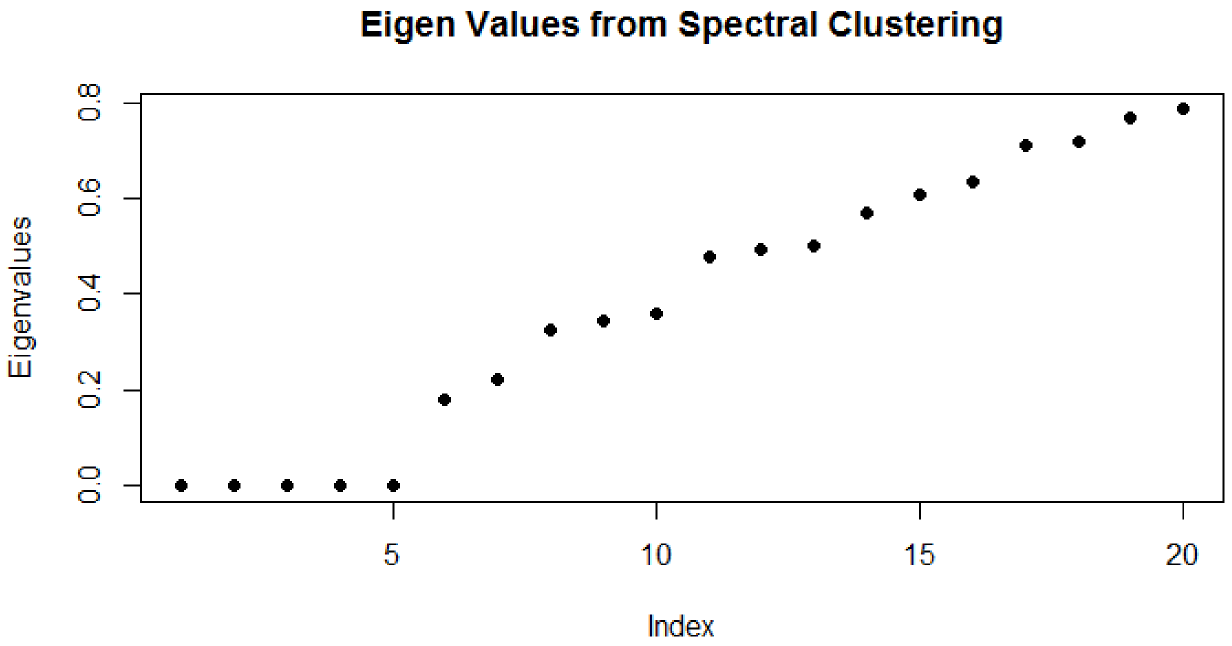

Supplementary Figure 10. Spectral clustering confirms five clusters identified in the hierarchical clustering dendrogram. The plot shows that the multiplicity of the eigenvalue zero of the Laplacian matrix in the spectral clustering setting is five, suggesting that the number of clusters/subpopulations in primary tumor 0903 and 0909 combined data is five. This finding supports the 5 clusters visually identified in the hierarchical clustering dendrogram (Figure 4d).

Supplementary Table 1. Clinical-pathological characteristics of breast tumor biopsies.

Patient	ER/PR/HER2 IHC status	HER2 FISH	Path report	Number of panCK+ (R^2>0.7, SNR>5)	Number of HER2+ (R^2>0.7, SNR>5)	Number of t-erbB2 (R^2>0.7, SNR>10)	t-HER2+ /HER2 expression per t-erbB2 expressing cell	t-erbB2/HER2 expression in the whole tissue
0909	HER2(3+)<br>ER(3+)<br>PR(1-3+)<br>Ki67 = 35%	Positive<br>HER2:CEP17=<br>4.55	T3N2A	101	90<br>(89%)	36<br>(40%)	1 - 14%<br>(7.1±3.1%)	5.2%
0903	HER2(3+)<br>ER(-)<br>PR(-)<br>Ki67 = 60%	Positive<br>HER2:CEP17=<br>5.21	T1cN0	228	127<br>(56%)	19<br>(15%)	9 - 47%<br>(23.8±10.0%)	3.0%
0119	HER2(3+)<br>ER(3+)<br>PR(1+)<br>Ki67 = 15-20%	Positive<br>HER2:CEP17 =<br>2.78	pT2(m) pN0	54	36<br>(67%)	0	NA	NA
0301	HER2(3+)<br>ER(1-2+)<br>PR(-)<br>Ki67= 20-30%	np	pT2 pN1a	31	10<br>(32%)	0	NA	NA
1216	HER2(neg)<br>ER(3+)<br>PR(3+)<br>Ki67 = 10-15%	Negative	LN<br>infiltrated<br>T4b N1A	461	42<br>(9%)	0	NA	NA
1217	HER2(2+)<br>ER(1+)	Negative,<br>HER2:CEP17=	LN<br>infiltrated	96	6<br>(6%)	0	NA	NA

	PR(-)<br>Ki67 = 40-50%	1.64	T4b N1a					
0225	HER2(neg, 1+)<br>ER(3+)<br>PR(1+)<br>Ki67 < 5%	negative	pT2(m)N0	437	15<br>(3%)	0	NA	NA
Male	ER(3+), 95%<br>PR(2+)<br>Ki67 = 5-10%	Positive	ypT1a	12<br>(39%)	4<br>(33%)	0	NA	NA

Supplementary Table 2. Analysis of primary cells from tumor 0903 in the microwells.

Image	No. of microwell	Occupied microwell	No. of cells inside the microwell	No. cells in the image
1	2	0	0	0
2	2	0	0	0
3	0	0	0	0
4	2	1	1	3
5	2	0	0	2
6	2	0	0	2
7	2	0	0	5
8	2	0	0	3
9	2	1	1	4
10	0	0	0	3
11	2	1	1	3
12	2	1	1	10
13	2	0	0	0
14	2	0	0	0
15	0	0	0	1
16	2	0	0	0
17	2	0	0	0
18	2	0	0	5
19	2	0	0	0
20	2	0	0	3
21	2	0	0	7
22	0	0	0	3
23	2	0	0	4
Image	Total microwell	Occupied microwell	No. of cells inside the microwell	No. cells in the image
24	2	0	0	11
25	2	0	0	1
26	2	0	0	0
27	0	0	0	2
28	2	0	0	0
29	2	0	0	0
30	2	0	0	2
31	2	0	0	3
32	2	1	1	5
33	2	0	0	2
34	0	0	0	5
35	0	0	0	5
36	2	1	1	5
37	1	0	0	0
38	2	0	0	1
39	1	1	1	1
40	2	0	0	1
41	2	0	0	1
42	2	0	0	0
43	1	0	0	1
44	2	1	1	5
45	2	0	0	3
46	0	0	0	3
Image	Total microwell	Occupied microwell	No. of cells inside the microwell	No. cells in the image
47	2	0	0	4
48	2	1	1	8
49	2	0	0	0
50	2	0	0	0
	Total microwells	Total occupied microwells	Total No. of settled cells	Total No. of cells
SUM	81	9	9	127
		Microwell occupancy		Cell settling efficiency
%		11%		7%
